# Onset of Parkinson’s Disease Identified Through Hyperhidrosis: A Middle-Aged Woman Case Report

**DOI:** 10.3390/reports9010050

**Published:** 2026-02-02

**Authors:** Mirko Zitti, Alessandro Andreani, Daniele De Patre, Luisa Cacciante, Giorgia Pregnolato

**Affiliations:** 1 Department of Human Neuroscience, University of Rome La Sapienza, 00185 Rome, Italy; 2 IRCCS Ospedale Policlinico San Martino, 16132 Genoa, Italy; 3 Alma Mater Europea Center of Maribor, 2000 Maribor, Slovenia; 4 Independent Researcher, 19033 Castelnuovo Magra, Italy; aless39@gmail.com; 5 NeuroRiab Specialist Center for Post-Stroke and Neurological Rehabilitation, 64100 Teramo, Italy; danieledepatre1@alice.it; 6 Laboratory of Healthcare Innovation Technologies, IRCCS San Camillo Hospital, 30126 Venice, Italy; luisa.cacciante@hsancamillo.it; 7 Research Ireland Centre Insight, University College Dublin, Dublin 4, D04 V1W8 Dublin, Ireland; giorgia.pregnolato@ucd.ie

**Keywords:** Parkinson disease, cervical radiculopathy, hyperidrosis, screening for referral, case report

## Abstract

**Background and Clinical Significance**: Parkinson’s disease (PD) is a neurodegenerative condition characterized by motor and non-motor symptoms, which significantly impact patients’ autonomy and quality of life levels. Basically, the PD diagnosis is clinical and, in some cases, can be challenging to diagnose due to the heterogeneity of the symptoms. **Case Presentation**: A 58-year-old woman who, during the COVID-19 lockdown, referred to experiences of slight tremor and stiffness in her left hand at rest, but without any other associated symptoms. Firstly, after consulting a general practitioner (GP), the patient was diagnosed with cervical radiculopathy (CR), presented as essential tremor and stiffness to the hand. Nevertheless, during the initial physiotherapy evaluation, the motor symptoms did not fully align with the diagnosis of CR. For this reason, the presence of non-motor symptoms was thoroughly investigated. Notably, hyperhidrosis was identified as a significant non-motor symptom, leading to the patient’s subsequent referral to a neurologist, who finally diagnosed PD. **Conclusions**: This case report highlights the essential role of physiotherapists in conducting independent assessments and comprehensive investigations of all patients’ symptoms, even when a medical diagnosis has already been established. This is particularly crucial when there is suspicion that musculoskeletal symptoms may be indicative of neurodegenerative diseases such as PD, which is well-known for its extensive array of non-motor symptoms. Especially in women with PD, non-motor symptoms tend to emerge earlier and in a more subtle manner than motor symptoms, making diagnosis challenging. Therefore, meticulous anamnestic data collection is essential, especially by physiotherapists working in direct-access settings.

## 1. Introduction and Clinical Significance

Parkinson’s disease (PD) is the second most common neurodegenerative disorder after Alzheimer’s [1]. Nowadays, more than 6 million people are affected by this disease worldwide, and in Western Europe alone, there are more than 800,000 people with a diagnosis of PD [1].

The prevalence of the disease is higher in men than in women, although mortality rates are higher among women [2].

A key feature of PD is the loss of dopamine-producing neurons in a part of the brain called the substantia nigra pars compacta. In particular, the ventrolateral tier, whose neurons send signals to the dorsal putamen, is the area most affected. Degeneration of this nigrostriatal pathway leads to reduced dopamine signaling in the sensorimotor striatum, altering the balance of basal ganglia circuits and disrupting normal motor processing [1,3,4].

Research has shown that the loss of dopaminergic neurons in the substantia nigra pars compacta is closely associated with the emergence of cardinal motor symptoms, including bradykinesia and muscular rigidity. More recent evidence indicates that this neuronal degeneration begins early in the course of the disease, prior to the onset of clinically overt motor manifestations, and that a proportion of dopamine-producing neurons may remain functionally viable and potentially recoverable during the initial stages of PD [1].

Parkinson’s disease, however, is not restricted to the involvement of the substantia nigra. Multiple additional brain regions and neuronal systems are affected, including the locus coeruleus, the basal nucleus of Meynert, the pedunculopontine nucleus, the raphe nuclei, the dorsal motor nucleus of the vagus, the amygdala, and the hypothalamus [1].

Many people with PD also show what is known as Lewy pathology, abnormal accumulations of the protein α-synuclein, which form Lewy bodies inside nerve cells and Lewy neurites along their connections. Importantly, recent findings show that PD is more complex than just the formation of Lewy bodies and likely involves multiple biological processes, influenced in part by genetic factors [1,3,4].

In line with this growing complexity, recent studies suggest that PD may follow at least two distinct pathological trajectories, determined by the site of origin and propagation of pathological α-synuclein aggregates. In the “brain-first” subtype, α-synuclein pathology is thought to originate within the central nervous system and subsequently spread caudally, whereas in the “body-first” subtype, it initially arises in the peripheral autonomic nervous system, most notably the enteric nervous system, and ascends via the vagus nerve to the brainstem, leading to early autonomic dysfunction [5].

The presence or absence of rapid eye movement sleep behavior disorder (RBD) at motor onset has emerged as a key clinical marker distinguishing these trajectories. Body-first PD is typically associated with early RBD, greater peripheral α-synuclein burden, and more symmetrical dopaminergic degeneration, while brain-first PD more often shows asymmetric nigrostriatal involvement and distinct genetic associations.

Together, these models underscore the heterogeneity of PD and support the need for subtype-specific diagnostic and therapeutic approaches, particularly with respect to non-motor symptoms related to autonomic dysfunction [5]. For clinicians, an important part of diagnosis is distinguishing PD from other conditions that can look similar, including Progressive Supranuclear Palsy with predominant parkinsonism and Multiple System Atrophy, in which autonomic symptoms are often particularly prominent, especially in the early stages when these disorders may closely resemble PD [6].

Dementia with Lewy bodies should also be actively considered in the differential diagnosis, particularly in patients with early non-motor or autonomic symptoms, as it may closely mimic PD in the initial stages [7].

Regarding risk factors for PD, gender differences are evident [8]. For example, in men, risks include factors such as a lack of caffeine consumption, head trauma, and exposure to pesticides, while anemia emerges as a risk factor in women [9]. Conversely, caffeine consumption has shown a protective effect in PD among women [10].

Parkinson’s disease treatments include pharmacological, such as levodopa administration with or without other drugs, and non-pharmacological approaches, such as physical activity, physiotherapy, occupational therapy, and speech therapy [11,12,13].

Parkinson’s disease diagnosis is clinical and based mainly on the observation of core motor symptoms, such as stiffness, tremor, and bradykinesia [1]. In more advanced stages, motor symptoms may include chewing and speaking impairments, masked facial expressions, and postural instability [1].

Moreover, PD could be characterized by non-motor symptoms, such as depression, anxiety, emotional changes, cognitive impairments, swallowing, urinary problems, constipation, fatigue, sleep disturbances (i.e., RDB), olfactory dysfunction, orthostatic hypotension, and a spectrum of autonomic nervous system dysfunctions [3,4].

The characterization of sex-related differences in motor symptom patterns can play a crucial role in terms of diagnosis and therapeutic strategies [9]. Regarding the diagnostic process, women with PD experience delays in diagnosis and in specialist referral as compared to men with PD [10]. Moreover, in terms of therapeutic aspects, several differences exist in the type and timing of motor and non-motor symptom onset. Firstly, with respect to motor symptoms, women tend to develop them later than men, with specific characteristics such as reduced mobility and tremor (i.e., the first and most common symptom). Women also show a tendency to develop postural instability, which causes a greater risk of falls and a high risk of motor complications due to levodopa [13]. In contrast, the freezing of gait and camptocormia are common symptoms observed in men [8,9].

Secondly, concerning non-motor symptoms, women reported higher rates of fatigue, depression, anxiety, constipation, restless leg syndrome, and pain, while exhibiting lower rates of anosmia, autonomic dysfunction, apathy, and rapid eye movement sleep behavior disorder than men [10]. Furthermore, an association between female gender and pain has been established. Indeed, the female gender acts as a predictor for the overall severity of pain, along with affective and autonomic symptoms and motor complications [9].

Many of these symptoms appear just before the age of 50 but are often not recognized, so most patients receive a later diagnosis [14]. Indeed, the presence of non-motor symptoms (e.g., hyperhidrosis) is not typically detected in the early stages of PD, whereas motor symptoms in PD could be confusedly interpreted as any other musculoskeletal disorder, such as radiculopathy [15].

Cervical radiculopathy (CR) is a neck disorder caused by the compression or irritation of nerve roots in the cervical spine [16]. Radicular pain is the most common symptom, followed by paresthesia. The distribution of paresthesia and pain usually follows the dermatomal distribution [16,17]. Currently, there is no gold standard for the diagnosis of CR, but diagnostic tools such as X-rays, magnetic resonance imaging (MRI), computed tomographic myelography, or electrodiagnostic testing are commonly performed [18,19]. The foraminal compression test, or Spurling’s test, is the best clinical test for confirming the diagnosis of CR [18,20].

Recent epidemiological studies have reported no significant sex differences in the prevalence, incidence, and years lived with disability across age groups in patients with neck disorders (NDs) [21]. However, the ND prevalence is consistently higher in women across all age groups. Recent literature indicates that psychological factors (e.g., stress, certain cognitive factors, and sleep problems) and individual/biological factors (e.g., preexisting neuromuscular or autoimmune disorders, aging, and genetic factors) contribute to the development of ND [21]. Work-related factors, such as working in awkward or sustained postures, play a role in ND development as well [21].

In the management of patients with CR, motor rehabilitation is necessary to reduce pain and disability [19]. Specifically, physical assessment aims to find common signs, such as neck and arm pain [18]. The pain is described in a range from light to severe burning pain [18]. Nevertheless, a small number of patients may present only weakness, without significant pain or sensory complaints [16,17,18]. Treatment is typically conservative, involving physiotherapy and anti-inflammatory medications [16,17].

The purpose and the novelty of this case report are to discuss a screening procedure for referral conducted through a comprehensive examination of non-motor symptoms that are not typically detected in the early stages of PD (e.g., hyperhidrosis). This was performed despite the patient already having a medical diagnosis of radiculopathy. The case aims to support physiotherapists working in direct access, particularly when managing patients in the early stages of disease. Early PD symptoms may overlap with those of neurological or musculoskeletal disorders, making it more difficult to differentiate.

## 2. Case Presentation

**Figure 1:**
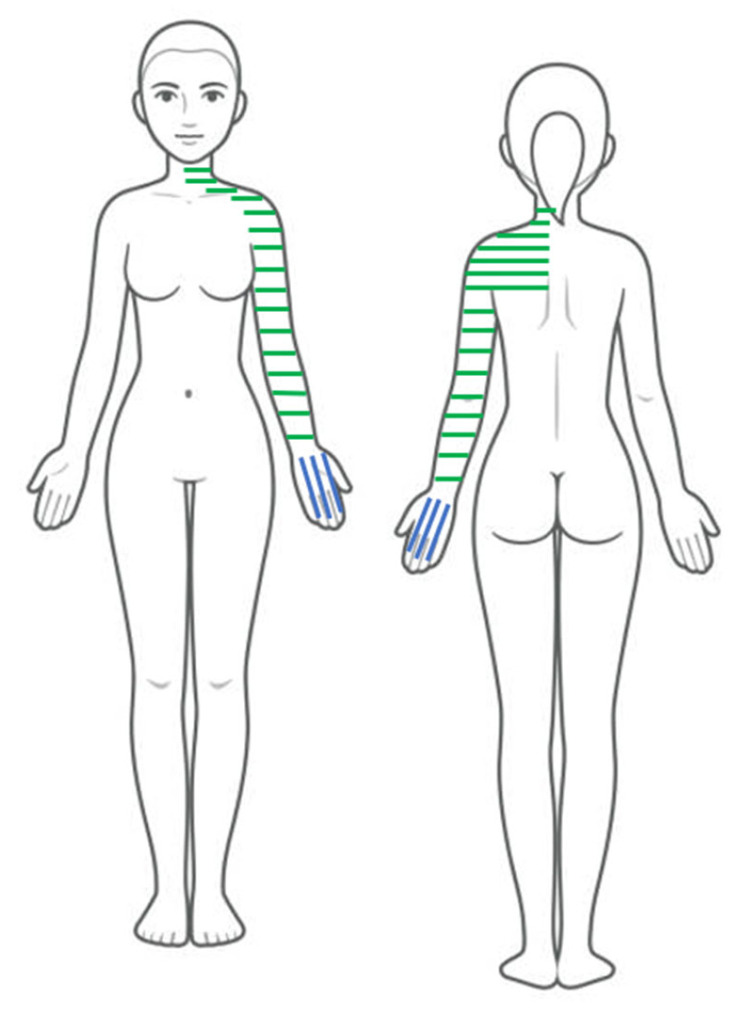
Body chart of the patient’s motor symptoms. Note: Blue lines represent tremor; green lines represent stiffness.

A 58-year-old female teacher was referred to physiotherapy by her general practitioner (GP) with a diagnosis of CR. The patient’s initial symptoms included a resting tremor in her left hand (i.e., the first symptom) and a sensation of stiffness in the musculature of the left deltoid, trapezius, scalene, and sternocleidomastoid muscles (Figure 1). The GP attributed the tremor to an idiopathic essential tremor [22], for which no medication had been recommended.

The patient reported that, during the national lockdown of the COVID-19 pandemic in March 2020, she began experiencing a resting tremor in her left hand. She thought it was a consequence of the anxiety induced by the pandemic. Another symptom she reported was the stiffness of all the muscles of the left shoulder (i.e., the left deltoid, trapezius, scalene, and sternocleidomastoid). Additionally, she noted that this tremor intensified during particular emotionally distressing situations.

The patient required medical attention approximately four months after the onset of the initial symptoms, but she attended physiotherapist one year after the initial medical diagnosis. Her primary concern was the absence of spontaneous improvement over this extended period.

She began physiotherapy based on her GP’s earlier recommendation a year later.

### 2.1. Medical History

In her remote medical history, the patient reported no family of neurodegenerative diseases, no history of trauma or significant past medical conditions, and no history of smoking or alcohol abuse. Grade 1 arterial hypertension was reported and was under treatment. She reported experiencing episodes of cervical pain that occasionally radiated to the arm but without associated tremor. These episodes resolved spontaneously. No episodes of postural instability, either static or dynamic, nor generalized fatigue were reported. The patient denied any sleep disturbances or alterations in sleep–wake rhythms. Functionally, she reported a good quality of life, with no limitations in Activities of Daily Living (ADL) and work-related tasks. The patient did not report any level of disability, even during complex or highly functional activities, such as manual tasks or repetitive hand movements.

### 2.2. First Diagnostic Imaging

To confirm the diagnosis of CR, during his medical examination, the GP prescribed the following diagnostic tests: cervical spine MRI and surface electromyography (EMG) of both upper limbs. The results were as follows:

- Cervical spine MRI: The examination revealed disk protrusions with a midline posterior orientation at the level between C3 and C4, C5 and C6, and a marginal direction in C6 and C7. Moderate somatic and facet joint arthritic changes were observed. No bone edema was detected, and spinal canal maintained normal width.
- EMG data showed findings involving the median and ulnar nerves in the right and left deltoid, biceps, triceps, and dorsal interosseous muscles. The findings were consistent with chronic radiculopathy on the right side at C5–C6, without signs of active denervation. Chronic radiculopathy on the left side at the levels C5–C6–C7 was mild, with no signs of denervation in progress.

The tests were performed one week after the initial medical consultation, which occurred four months after symptom onset.

### 2.3. Clinical Examination

One year after receiving the diagnosis of CR from GP, the patient presented to the physiotherapist clinic.

In the first session, the physiotherapist conducted a comprehensive clinical examination. From observation, no postural alterations or cervical spine deformities were observed. The physical examination did not reveal any deficits in strength or balance, no coordination differences compared to the right side [3,23]. Mobility at cervical and thoracic spine level was preserved. During both passive and active mobilization, no resistance was detected at varying speeds, indicating no increase in muscle tone. The patient showed a normal neurological examination, with full range of motion, preserved deep tendon reflexes, and normal sensory testing comparable to the contralateral side [3,23].

The patient retained preserved facial mimicry as well as pendular movements [3,23].

To verify the medical diagnosis of radiculopathy, the Wainner’s cluster [20] was performed, yielding negative results [20]. The Wainner cluster comprises four tests used to identify the presence of a radiculopathy. The four tests are Upper Limb Neurodynamic Test 1 (ULTT1), which involves cervical rotation less than 60°, distraction, and the Spurling test. If a patient has positive results for three out of four, the probability of the presence of radiculopathy increases to 65%, and if all four variables are present, the probability increases to 90% [20].

The only findings during the execution of ULNT1 [24] was mild resistance to mobilization on the affected side compared to the contralateral limb.

Other neurodynamic tests—ULNT2a, ULNT2b, andULNT3a [24]—performed for the upper limb were negative for rigidity and tremor induction, which were the main symptoms referred by the patient [25].

The Disabilities of the Arm, Shoulder, and Hand questionnaire (DASH) score was 1.47%. The DASH questionnaire was developed to measure physical disability and symptoms of the upper extremities in people with upper extremity disorders (hand, wrist, elbow, and shoulder) [26].

Notably, no tremor was observed or reported to have onset at any point during the assessment.

During the clinical examination, potential autonomic symptoms were also investigated. The patient reported excessive sweating at rest and during the night (she was not under any medication and had already gone through menopause), coinciding with the onset of tremor and rigidity. No dysregulation of thermoregulation was reported by the patient or observed on examination. This symptom had not been present before the appearance of tremor and rigidity, and it had not been explored by the GP during the initial medical history. The patient had not reported it spontaneously, as she did not consider it relevant to her condition.

### 2.4. Screening for Referral

After the clinical assessment, the diagnosis of CR was found to be inconsistent with the overall findings because the specific diagnostic criteria for CR or radicular pain were not fully met [18].

Although abnormalities were detected on MRI and EMG performed after the GP’s examination, such findings are frequently observed in asymptomatic individuals [27]. Therefore, in the absence of appropriate clinical presentation, these results alone cannot be considered sufficient to attributable solely to these findings [27,28].

Thus, considering that the neurological examination provided negative results, and the balance and coordination tests were also negative, and no restrictions in ADL and work activities were reported by the patient, further investigation into potential non-motor symptoms was deemed necessary.

Particularly, episodes of hyperhidrosis were reported by the patient in the last year. Indeed, as noted by Metzge et al. [29], this is one of the non-motor symptoms that may occur in PD [4,29].

Furthermore, considering the resting tremor associated with arm rigidity, the negative results from the various cervical assessment test, and the onset of hyperhidrosis, the patient was referred to a neurologist, in contrast with the GP’s suggestion to treat the tremor as an essential in nature.

Considering that the patient is at the lower end of the typical age range for disease onset, and the anxiety she exhibited during the evaluation regarding a potential diagnosis of PD, the patient was reassured and prepared [30] before meeting the neurologist to confirm or exclude a diagnosis of PD or parkinsonism.

**Figure 2:**
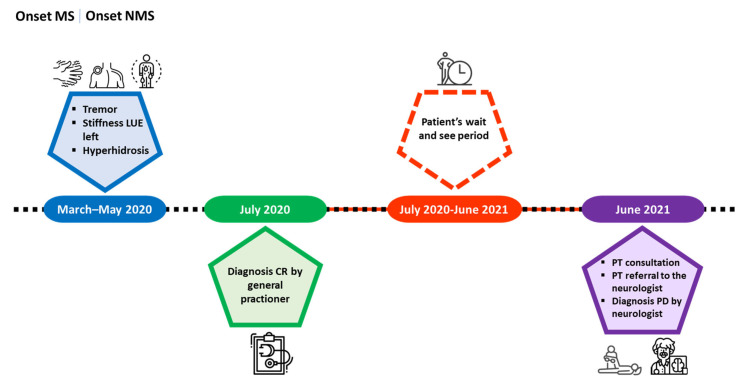
The timeline illustrates the comprehensive process that guided the patient from the initial CR diagnosis to the conclusive PD diagnosis, facilitated through the referral by the physiotherapist. Abbreviations: LUE: left upper extremity; MS: motor symptoms; NMS: non-motor symptoms; CR: cervical radiculopathy; PD: Parkinson’s disease; PT: physical therapist.

For a comprehensive overview of the entire course, refer to the timeline (Figure 2).

### 2.5. Second Diagnostic Imaging

As expected, after the neurological medical examination, the patient received a diagnosis of PD. As a first step, a brain MRI was prescribed for the patient to rule out other significant neurological pathologies.

Brain MRI revealed multiple bilateral gliotic–lacunar area appearances in the cortico-subcortical site and deep white matter, predominantly in the radiated crowns, which suggested a possible chronic angiopathy. No expansive lesions were evident in the cerebellar bridge or corners. There were no hemosiderin deposits or areas of focal pathological signal restriction in the diffusion-weighted imaging of 285 sequences related to recent ischemic events in the cytotoxic edema phase. All MRI images are available in Supplementary Materials File S1.

### 2.6. Treatment

After the neurological medical clinical assessment, conducted by a neurologist specialized in PD, the patient was diagnosed with idiopathic PD, characterized by unilateral onset and tremor, consistent with a tremor-dominant phenotype [31].

The patient was rated Hoehn Yahr (H&Y) stage 1 [32]. The Hoehn Yahr scale is designed to be a descriptive staging scale that provides a general estimate of clinical function in PD, combining functional deficits (i.e., disability) and objective signs (i.e., impairment) [32].

A score of 1 indicates unilateral involvement only, typically characterized by motor symptoms such as resting tremor, rigidity, and/or bradykinesia affecting a single side of the body, without impairment of balance or postural reflexes [32]. In the present case, the H&Y stage was assigned based on the presence of unilateral motor symptoms and the absence of axial involvement or postural instability during the neurological medical clinical assessment.

The Movement Disorder Society Unified Parkinson’s Disease Rating Scale (MDS-UPDRS) scale score was 10 [33]. The MDS-UPDRS is a tool to assess the patient’s level functional dependency in basic activities of daily living [33]. The total MDS-UPDRS score was derived from the standardized assessment of items across all domains of the scale, including mentation, behavior, and mood (Part I); functional performance in basic activities of daily living as reported by the patient (Part II); objective motor examination performed by the clinician (Part III); and complications of therapy, such as dyskinesias and motor fluctuations (Part IV). Each item was rated according to established criteria on a five-point ordinal scale ranging from normal (score of 0) to severe impairment (score of 4), with higher scores reflecting greater symptom severity and functional impact [33]. The score in this patient reflects mild disease severity, with low-impact symptoms predominantly affecting motor function and daily activities. The low overall score indicates functional independency and preserved autonomy.

The patient’s motor function, assessed by the UPDRS Part III, yielded a score of 6. For the detailed scoring of all MDS-UPDRS sections, including the individual subscores of each component and the H&Y staging, see Supplementary Materials File S2.

Considering the tremor onset, in accordance with study by Cerri et al. and Haxaam et al. [8,9], it was deemed one of the most benign forms of the phenotype [8,9].

Pharmacological therapy was initiated with 100/25 mg of levodopa/benserazide administered twice daily. Moreover, 0.314 mg of pramipexole once daily was added as adjunctive therapy. An amount of 5 mg of selegiline hydrochloride once daily was prescribed for 15 days and subsequently discontinued, after which by 100 mg of safinamide once daily was introduced following discontinuation of selegiline [19].

Additionally, the patient was addressed to resume physiotherapy and engage in physical activities, such as dancing, to effectively manage and minimize any potential complications that may arise in the future [12]. Physiotherapy sessions lasted one hour and were structured with aerobic training on the treadmill, incorporating auditory and visual cues [34]. Additionally, active coordination exercises involving both upper and lower limbs were included, ranging in intensity from moderate to intense [34,35]. The patient was advised to perform aerobic activities (such as walking or cycling) as part of her self-management plan at least 2–3 times a week, with each session lasting between 20 to 60 min [36].

### 2.7. Outcomes and Follow up

Subsequent to the PD diagnosis, the patient underwent an annual follow-up examination with the neurologist. At the present, four years after diagnosis, the patient has continued the prescribed drug therapy and engaged in weekly physiotherapy sessions. Her condition has remained stable, with no impairments in ADL or in regular work activities. The MDS-UPDRS was stable at 10 [33] and the DASH questionnaire score was 1.47 [26].

### 2.8. Patient’s Perception

The patient has reported that the anxiety experienced during the diagnosis period of PD has currently dissipated, and she is no longer as concerned as before. Furthermore, she states that she is satisfied with her QoL, which has not changed compared to before the diagnosis. In fact, she reports an improved sense of well-being attributed to the alleviation of stiffness and a reduction in resting tremor through the implementation of physiotherapy and pharmacological treatment.

## 3. Discussion

Parkinson’s disease, with its subtle onset and a diagnosis exclusively based on the clinical evaluation, can often be underestimated or confused with other conditions, thus delaying diagnosis and related treatment [3,4,9]. Specifically, many cases of early-stage PD are frequently mistaken for shoulder disorders and ND, leading patients to undergo treatments for musculoskeletal issues, as observed in this case [37,38]. In this case, the symptoms involving the shoulder and neck were primarily attributable to PD itself rather than to a coexistence of musculoskeletal pain and early PD manifestations. A study conducted by Stamey et al. demonstrates that 11% of PD patients reported experiencing shoulder pain before receiving a PD diagnosis [39]. Another study of Schrag et al. found a similar incidence of ND or stiffness in individuals with subsequent PD and controls. This similarity may be due to ND being a less specific symptom or not a pre-diagnostic feature of PD [39].

Concerning tremors, it is challenging to discern whether they are essential tremors or indicative of early-stage of PD, especially in young individuals [2,15,40,41]. Additionally, given the patient’s age is at the lower boundary of the typical onset range for PD (i.e., 65–70 years), it could easily lead to misinterpretation, raising suspicions of a musculoskeletal issue [37].

For these reasons, it is crucial to investigate non-motor symptoms, especially in women, where they may appear prodromic and may manifest up to ten years before the onset of PD [15].

Indeed, as noted by Haaxma et al. and Cerri et al., motor symptoms arise later in women than in men [8,9]. Therefore, a thorough exploration of autonomic, sensory, and neuropsychiatric non-motor symptoms could be more discriminating in the early recognition of PD [42].

Recent research has also highlighted the existence of distinct PD phenotypes, including brain-first and body-first subtypes [5]. In the present case, although the autonomic manifestation of hyperhidrosis was prominent and early, the patient’s overall clinical presentation does not allow for definitive classification into either of the proposed subtypes. Nevertheless, this observation reinforces a key principle emerging from the recent conceptualization of PD subtypes: even singular or subtle autonomic signs can constitute a valuable early clinical clue independent of the underlying pathological propagation pattern. This aligns with the understanding that autonomic involvement is a transversal and often early feature of PD, even if its extent and timing may vary between subtypes, as emphasized in the review by Horsager et al. [5]. Hyperhidrosis, while not among the most frequently investigated autonomic markers in subtype studies (which focus on constipation, orthostatic hypotension, and cardiac sympathetic denervation), represents a manifestation of sympathetic nervous system dysfunction [5]. Its early appearance in our case is consistent with the growing awareness that neurodegeneration affecting non-dopaminergic systems, including the autonomic nervous system, can precede and accompany the classical motor phase for a prolonged period, offering a critical window of opportunity for early diagnosis [5].

Therefore, even when a clinical presentation is not clearly attributable to a “pure” body-first phenotype, the presence of an autonomic symptom such as hyperhidrosis should not be overlooked. It underscores the importance of a holistic diagnostic approach that includes a careful non-motor assessment, as these signs may be among the first messengers of ongoing neurodegenerative pathology.

The uniqueness of this case lies not only in the prominence in hyperhidrosis as an early symptom [15] but also in the role of non-medical healthcare providers, specifically physiotherapists, in identifying these subtle clues. In direct-access settings, physiotherapists can contribute significantly to the early detection of both motor and non-motor symptoms, facilitating timely referral to neurologists when PD or other neurological disorders are suspected. This interprofessional collaboration represents a promising approach for early diagnosis and management, as physiotherapists’ observations can help identify underestimated symptoms, support prompt referral, and ultimately accelerate diagnostic decision making. In this case, a careful and meticulous anamnestic process, including attention to hyperhidrosis, proved crucial in guiding the patient toward an earlier diagnosis. However, through a meticulous anamnestic process, it turned out to be crucial for guiding the patient toward an early diagnosis.

Failure to recognize early onset can result in delayed diagnosis of PD [43]. Recent studies show that an early diagnosis and, consistently, an early treatment can help slow down the progression of the disease [43]. Both pharmacological and early rehabilitative interventions could potentially be neuroprotective and may assist in mitigating the disease’s progression [14,43].

While this case provides valuable insights, it is important to acknowledge certain limitations. The findings of this report are based on a single case, limiting the generalizability of the conclusions. Additionally, non-motor symptoms were assessed through clinical history and observation rather than standardized autonomic scales, which would allow for more objective and reproducible evaluation. Despite these limitations, this case underlines that systematic screening for autonomic and other non-motor symptoms should be considered in routine clinical and physiotherapy evaluations as part of early detection strategies.

This case highlights the importance for physiotherapists, especially those working with direct access, to thoroughly investigate not only motor symptoms but also non-motor symptoms when a serious condition such as PD could be suspected during the evaluation process. Attention to underestimated autonomic features, including hyperhidrosis, can provide crucial diagnostic clues that might otherwise be overlooked [4,29]. Combined with interprofessional collaboration, such careful evaluation can facilitate timely referral to neurologists and enable earlier diagnosis. Early recognition of autonomic non-motor symptoms, including hyperhidrosis, allows for the prompt initiation of both pharmacological and rehabilitative interventions, which may not only alleviate symptoms but also potentially slow disease progression [4,29].

## 4. Conclusions

This case underscores the vital role of physiotherapists in identifying early PD, especially when symptoms are mistaken for musculoskeletal disorders like CR. The key to a correct diagnosis was the investigation of non-motor symptoms, particularly hyperhidrosis, which contradicted the initial medical diagnosis.

A thorough assessment, including screening for subtle non-motor symptoms, is essential when a patient’s clinical presentation is atypical. This approach is crucial for facilitating timely neurological referral, ensuring an accurate diagnosis and enabling early intervention that can improve long-term patient outcomes.

## Data Availability

The original contributions presented in the study are included in the article. Further inquiries can be directed to the corresponding author.

## Supplementary Materials

The following supporting information can be downloaded at: https://www.mdpi.com/article/10.3390/reports9010050/s1, Supplementary Materials File S1: MRI images. Supplementary Materials File S2: MDS-UPDRS assessment. This case report was written following items on the CARE checklist [44]. A preprint has previously been published [45].

## Abbreviations

The following abbreviations are used in this manuscript:

PD	Parkinson’s disease
GP	General practitioner
CR	Cervical radiculopathy
MRI	Magnetic resonance imaging
LUE	Left upper extremity
MS	Motor symptoms
NMS	Non-motor symptoms
PT	Physical therapist
NDs	Neck disorders
ADL	Activities of Daily Living
EMG	Electromyography
ULNT	Upper Limb Neurodynamic Test
DASH	Disabilities of the Arm, Shoulder, and Hand Questionnaire
MDS-UPDRS	Movement Disorder Society Unified Parkinson’s Disease Rating Scale
H&Y	Hoehn and Yahr Scale
