# Supplementary material for: Onset of Parkinson’s Disease Identified Through Hyperhidrosis: A Middle-Aged Woman Case Report
Source: Reports (MDPI). 2026 Feb 2;9(1):50. doi: 10.3390/reports9010050 (PMC12922036; doi:10.3390/reports9010050)
Supplement: Supplementary file 1 [file reports-09-00050-s001.zip › CARE-checklist_PD.pdf]

CARE case report guidelines

CARE Checklist of information to include when writing a case report

(cc) BY-NC-ND

Topic	Item	Checklist item description	Reported on Line
Title	1	The diagnosis or intervention of primary focus followed by the words "case report"	1-3
Key Words	2	2 to 5 key words that identify diagnoses or interventions in this case report, including "case report"	35
Abstract (no references)	3a	Introduction: What is unique about this case and what does it add to the scientific literature?	14-17
	3b	Main symptoms and/or important clinical findings	18-26
	3c	The main diagnoses, therapeutic interventions, and outcomes	18-26
	3d	Conclusion—What is the main "take-away" lesson(s) from this case?	26-34
Introduction	4	One or two paragraphs summarizing why this case is unique (**may include references**)	104-110
Patient Information	5a	De-identified patient specific information.	113-129
	5b	Primary concerns and symptoms of the patient.	113-129
	5c	Medical, family, and psycho-social history including relevant genetic information	168-178
	5d	Relevant past interventions with outcomes	168-178
Clinical Findings	6	Describe significant physical examination (PE) and important clinical findings.	198-232
Timeline	7	Historical and current information from this episode of care organized as a timeline	260
Diagnostic Assessment	8a	Diagnostic testing (such as PE, laboratory testing, imaging, surveys).	179-196 / 275-282
	8b	Diagnostic challenges (such as access to testing, financial, or cultural)	238-260
	8c	Diagnosis (including other diagnoses considered)	285-289
	8d	Prognosis (such as staging in oncology) where applicable	293
Therapeutic Intervention	9a	Types of therapeutic intervention (such as pharmacologic, surgical, preventive, self-care)	295-305
	9b	Administration of therapeutic intervention (such as dosage, strength, duration)	295-305
	9c	Changes in therapeutic intervention (with rationale)	295-305
Follow-up and Outcomes	10a	Clinician and patient-assessed outcomes (if available)	308-312
	10b	Important follow-up diagnostic and other test results	308-312
	10c	Intervention adherence and tolerability (How was this assessed?)	308-321
	10d	Adverse and unanticipated events	
Discussion	11a	A scientific discussion of the strengths AND limitations associated with this case report	323-339
	11b	Discussion of the relevant medical literature **with references**.	327-331 / 340-350
	11c	The scientific rationale for any conclusions (including assessment of possible causes)	351-356
	11d	The primary "take-away" lessons of this case report (without references) in a one paragraph conclusion	358.365
Patient Perspective	12	The patient should share their perspective in one to two paragraphs on the treatment(s) they received	315-321
Informed Consent	13	Did the patient give informed consent? Please provide if requested	Yes ☒ No ☐
