# Supplementary material for: Onset of Parkinson’s Disease Identified Through Hyperhidrosis: A Middle-Aged Woman Case Report
Source: Reports (MDPI). 2026 Feb 2;9(1):50. doi: 10.3390/reports9010050 (PMC12922036; doi:10.3390/reports9010050)
Supplement: Supplementary file 1 [file reports-09-00050-s001.zip › Supplementary materials 2.pdf]

MDS UPDRS Score Sheet

1.A	Source of information	☑ Patient<br>☐ Caregiver<br>☐ Patient + Caregiver

Part I

1.1	Cognitive impairment	0
1.2	Hallucinations and psychosis	0
1.3	Depressed mood	0
1.4	Anxious mood	2
1.5	Apathy	0
1.6	Features of DDS	0
1.6a	Who is filling out questionnaire	☑ Patient<br>☐ Caregiver<br>☐ Patient + Caregiver
1.7	Sleep problems	0
1.8	Daytime sleepiness	0
1.9	Pain and other sensations	0
1.10	Urinary problems	0
1.11	Constipation problems	0
1.12	Light headedness on standing	0
1.13	Fatigue	0

Part II

2.1	Speech	0
2.2	Saliva and drooling	0
2.3	Chewing and swallowing	0
2.4	Eating tasks	0
2.5	Dressing	0
2.6	Hygiene	0
2.7	Handwriting	0
2.8	Doing hobbies and other activities	0
2.9	Turning in bed	0
2.10	Tremor	1
2.11	Getting out of bed	0
2.12	Walking and balance	0
2.13	Freezing	0
3a	Is the patient on medication?	☑ No ☐ Yes
3b	Patient's clinical state	☑ Off ☐ On
3c	Is the patient on levodopa?	☑ No ☐ Yes
3.C1	If yes, minutes since last dose:	//

Part III

3.1	Speech	0
3.2	Facial expression	0
3.3a	Rigidity– Neck	2
3.3b	Rigidity– RUE	0
3.3c	Rigidity– LUE	2
3.3d	Rigidity– RLE	0
3.3e	Rigidity– LLE	0
3.4a	Finger tapping– Right hand	0
3.4b	Finger tapping– Left hand	0
3.5a	Hand movements– Right hand	0
3.5b	Hand movements– Left hand	0
3.6a	Pronation- supination movements– Right hand	0
3.6b	Pronation- supination movements– Left hand	0
3.7a	Toe tapping– Right foot	0
3.7b	Toe tapping– Left foot	0
3.8a	Leg agility– Right leg	0
3.8b	Leg agility– Left leg	0
3.9	Arising from chair	0
3.10	Gait	0
3.11	Freezing of gait	0
3.12	Postural stability	0
3.13	Posture	0
3.14	Global spontaneity of movement	0
3.15a	Postural tremor– Right hand	0
3.15b	Postural tremor– Left hand	1
3.16a	Kinetic tremor– Right hand	0
3.16b	Kinetic tremor– Left hand	0
3.17a	Rest tremor amplitude– RUE	0
3.17b	Rest tremor amplitude– LUE	1
3.17c	Rest tremor amplitude– RLE	0
3.17d	Rest tremor amplitude– LLE	0
3.17e	Rest tremor amplitude– Lip/jaw	0
3.18	Constancy of rest tremor	0
	Were dyskinesias present?	☑ No ☐ Yes
	Did these movements interfere with ratings?	☑ No ☐ Yes
	Hoehn and Yahr Stage	1

Part IV

4.1	Time spent with dyskinesias	0
4.2	Functional impact of dyskinesias	0
4.3	Time spent in the OFF state	0
4.4	Functional impact of fluctuations	0
4.5	Complexity of motor fluctuations	0
4.6	Painful OFF-state dystonia	0
