# Supplementary figures and images for: Onset of Parkinson’s Disease Identified Through Hyperhidrosis: A Middle-Aged Woman Case Report
Source: Reports (MDPI). 2026 Feb 2;9(1):50. doi: 10.3390/reports9010050 (PMC12922036; doi:10.3390/reports9010050)

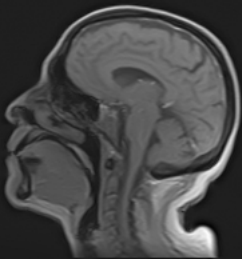

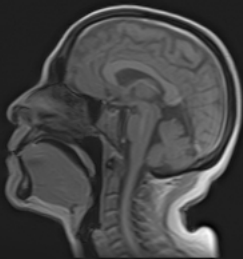

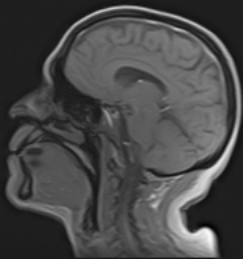

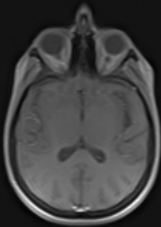

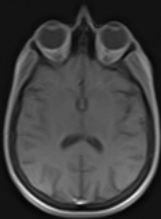

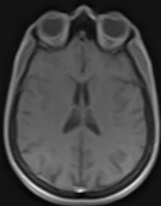

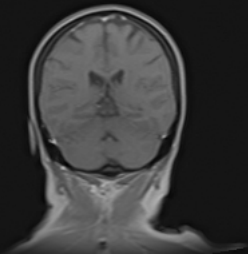

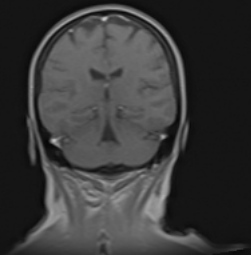

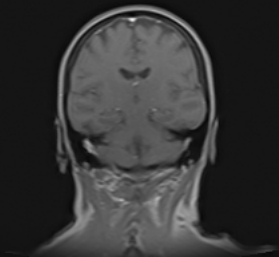

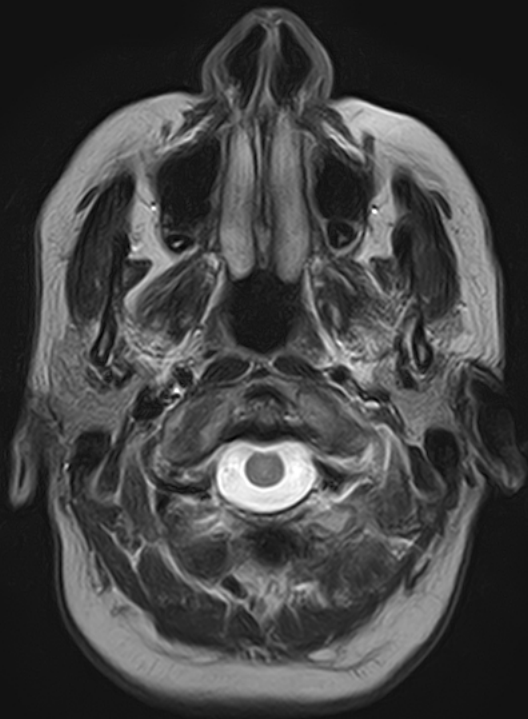

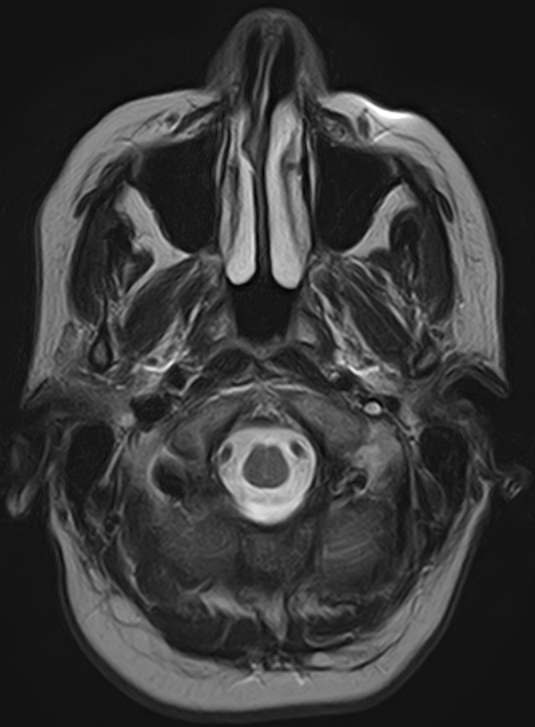

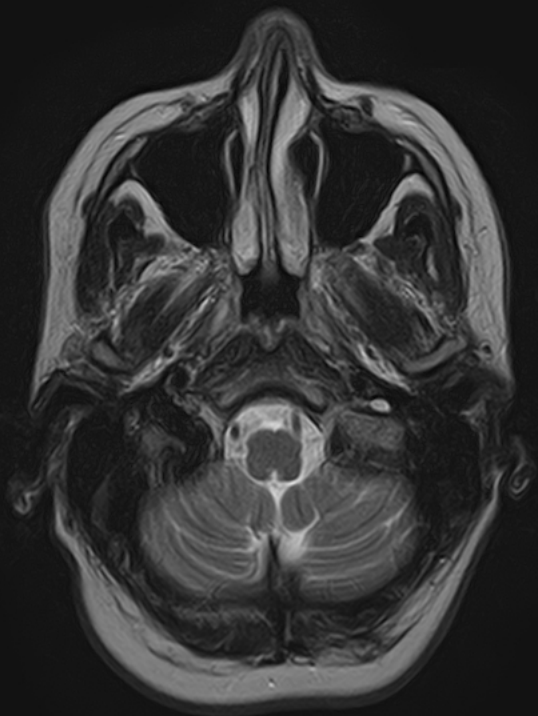

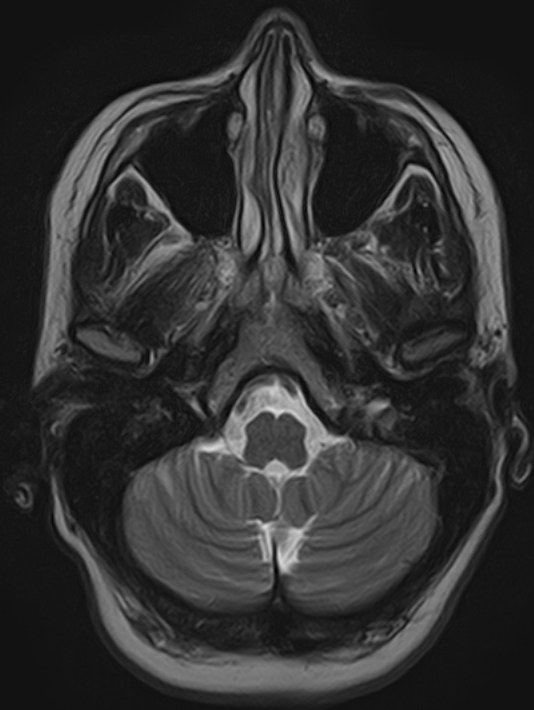

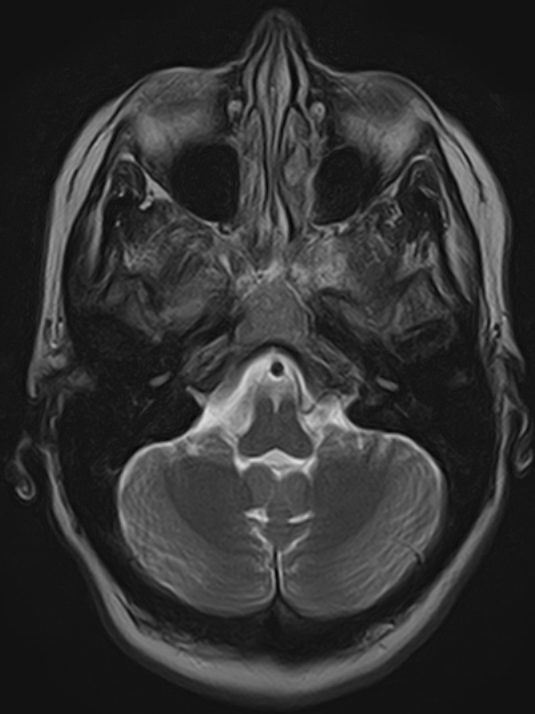

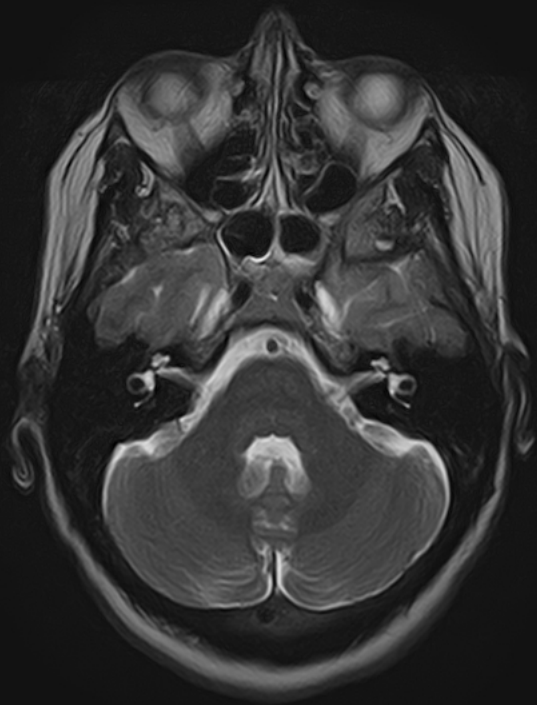

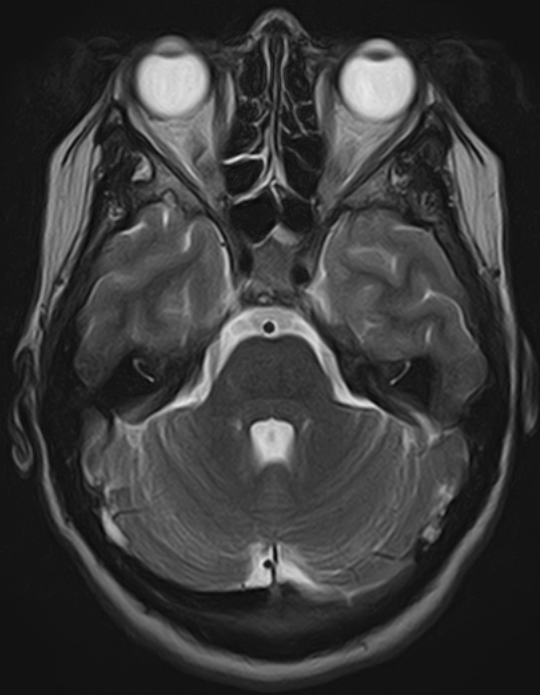

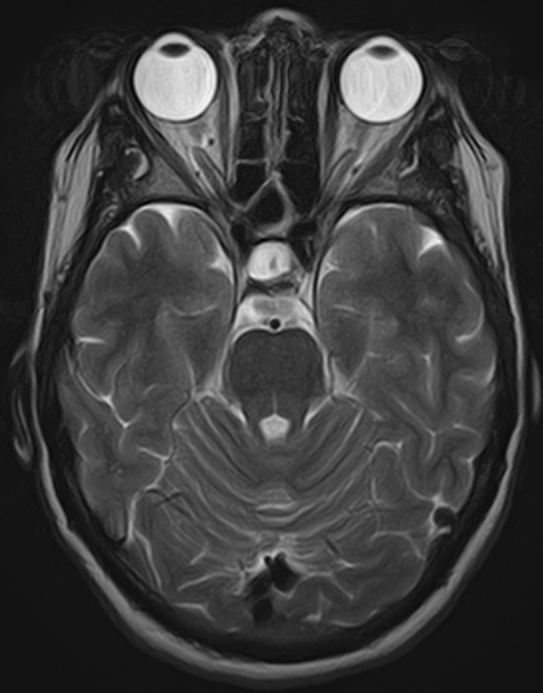

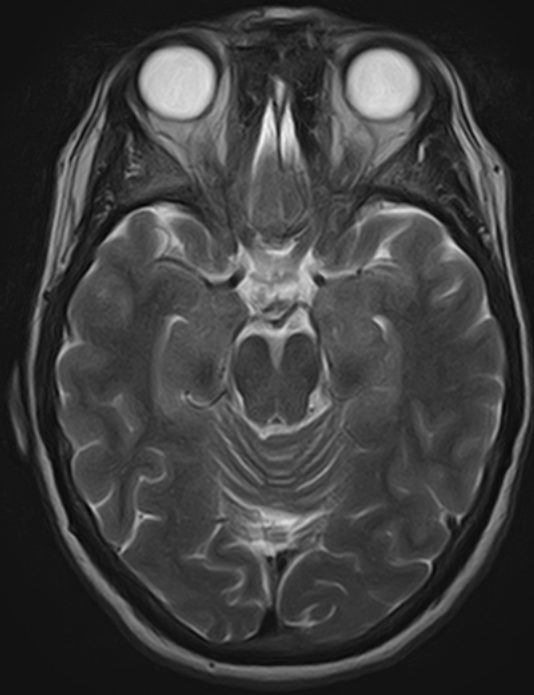

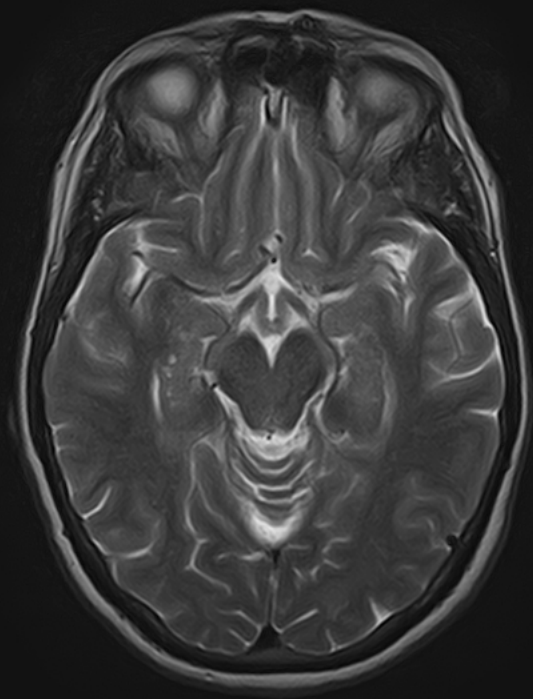

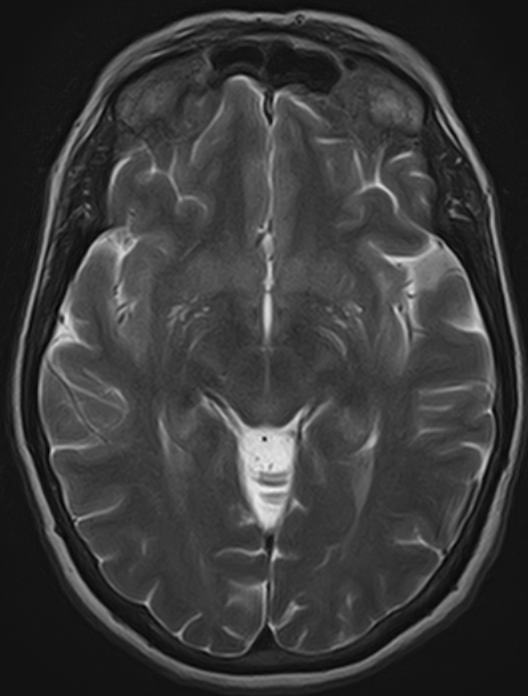

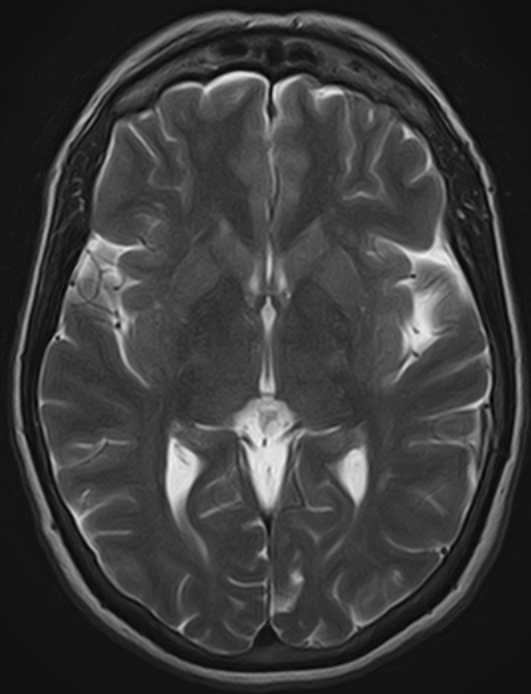

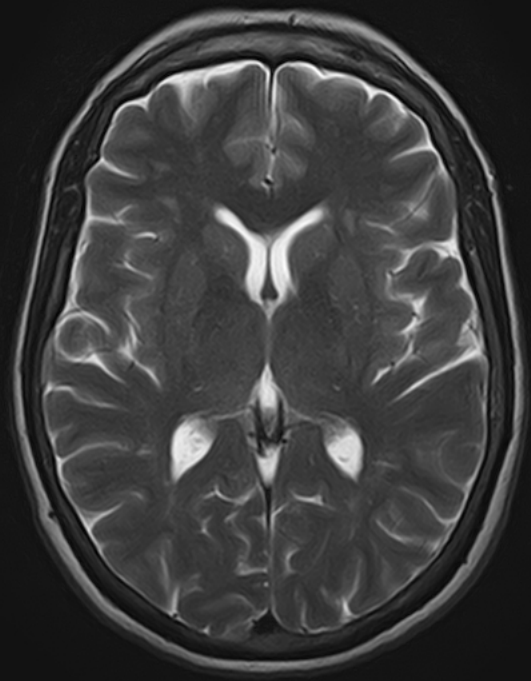

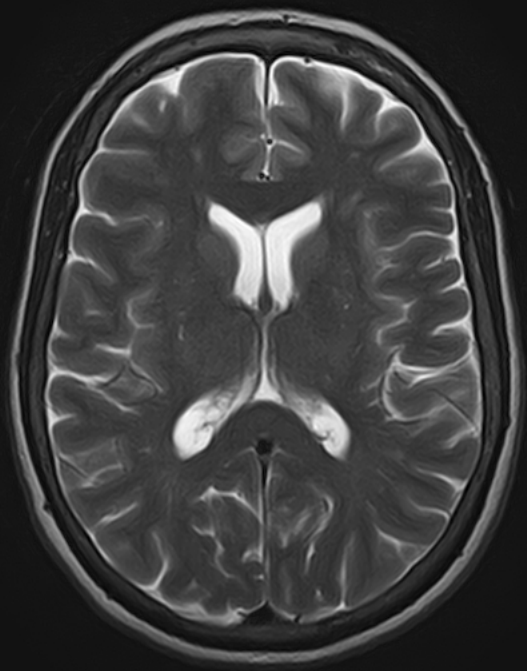

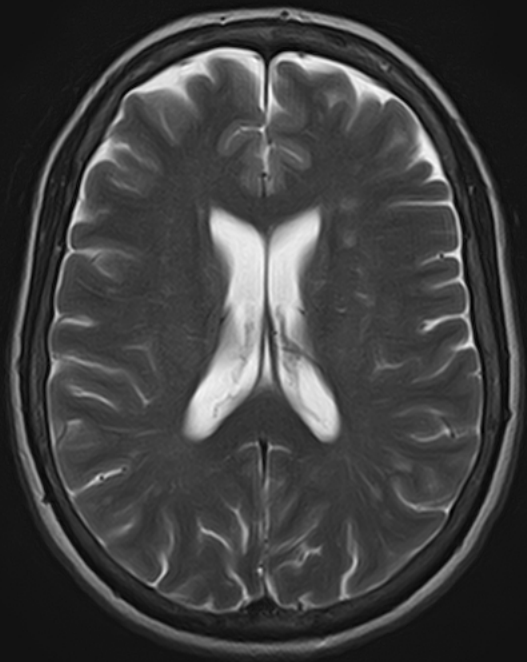

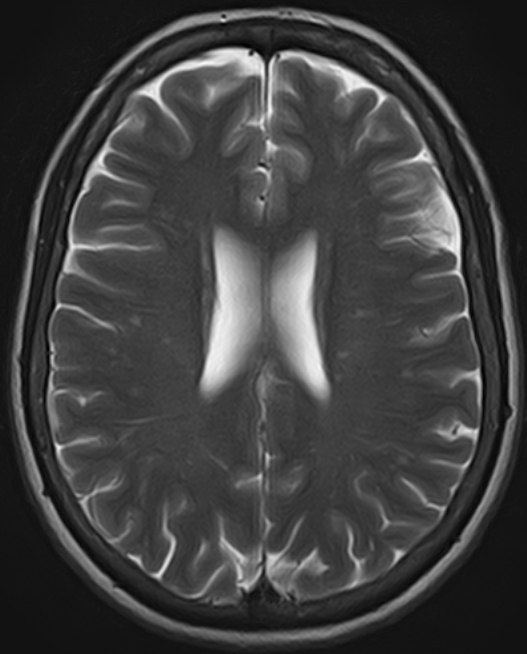

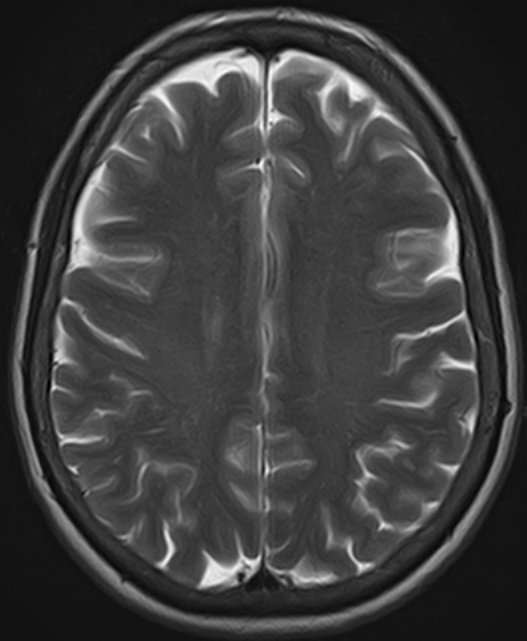

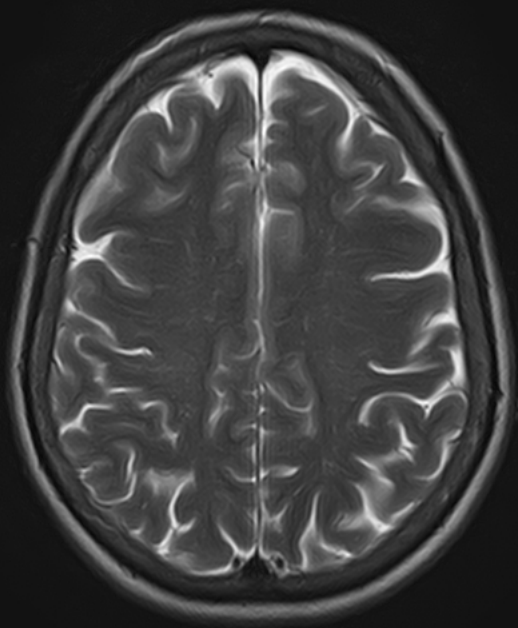

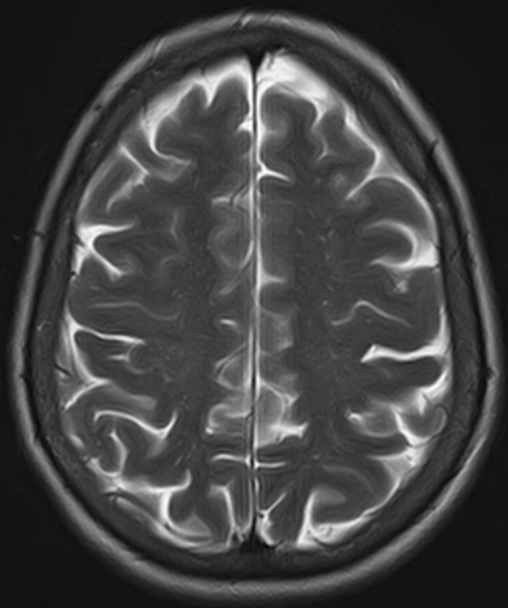

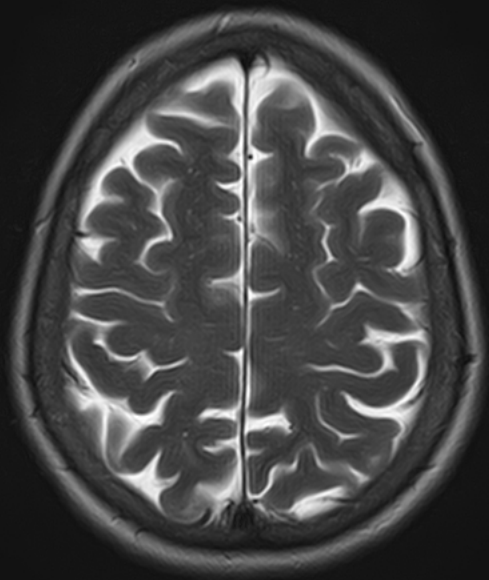

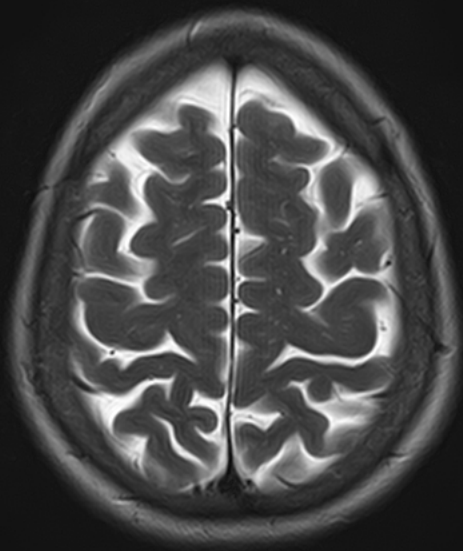

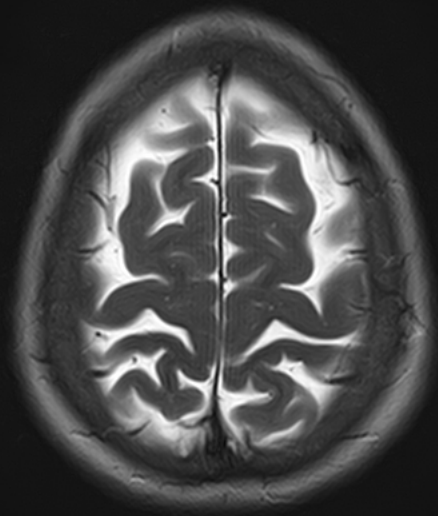

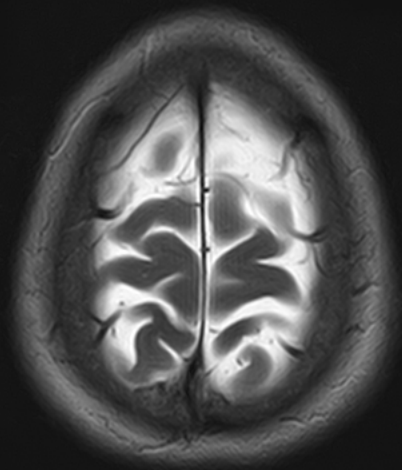

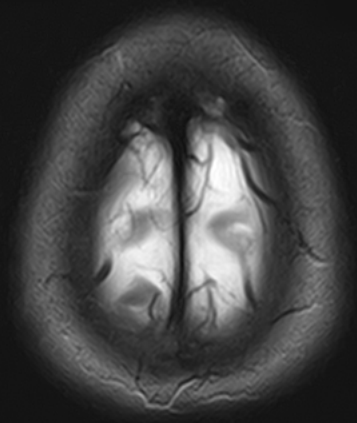

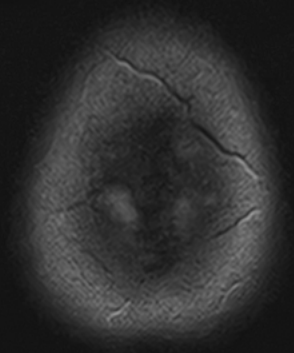

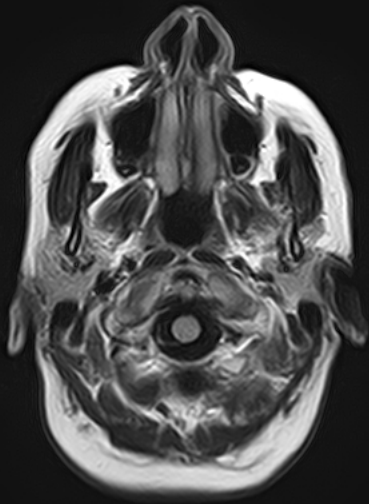

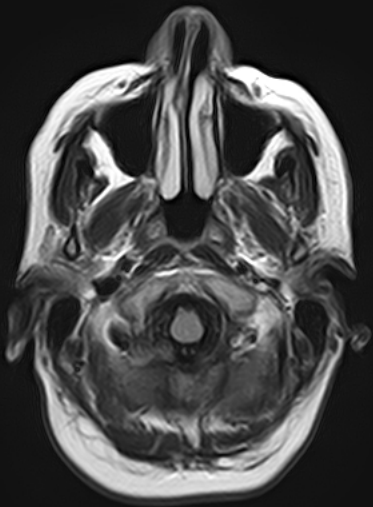

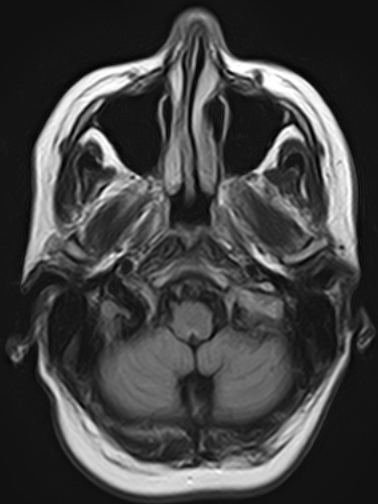

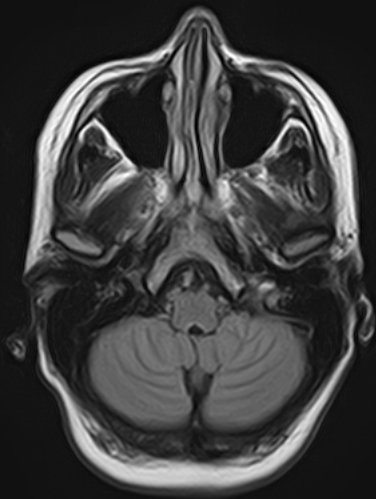

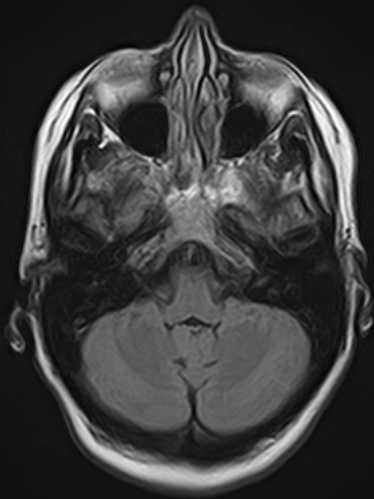

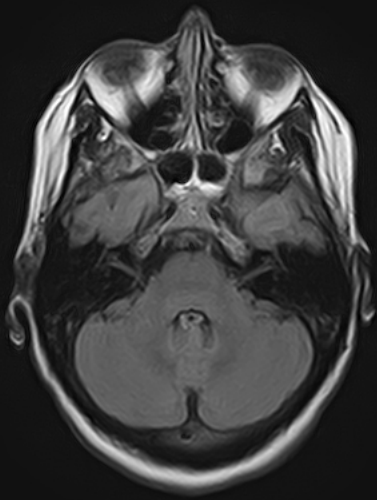

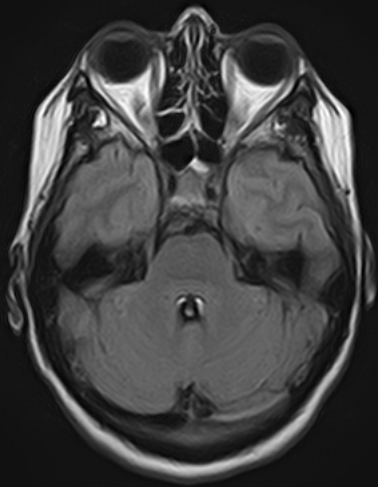

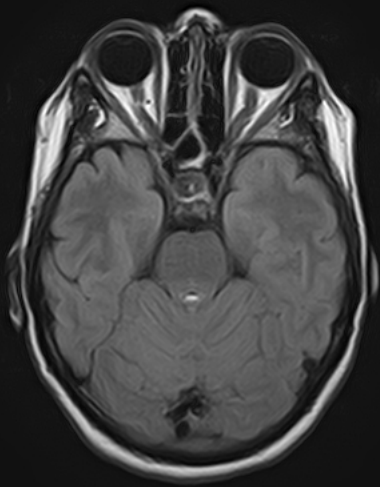

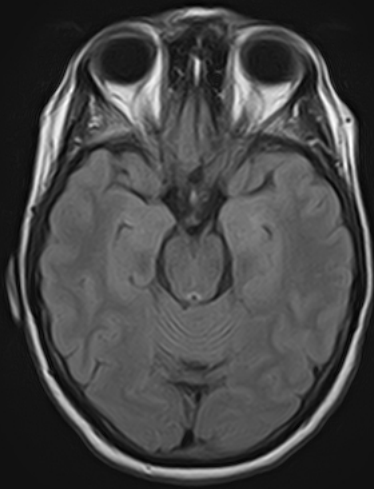

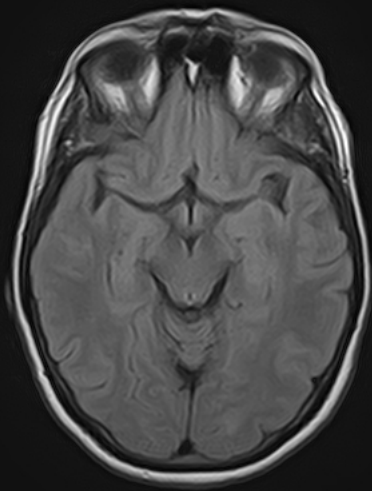

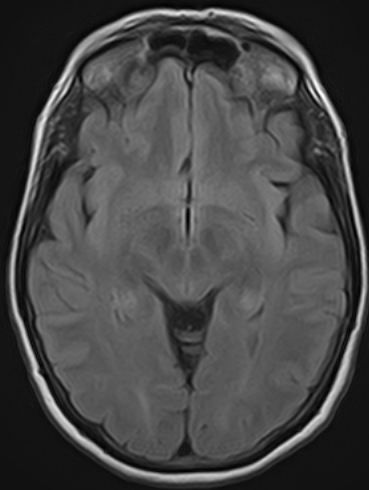

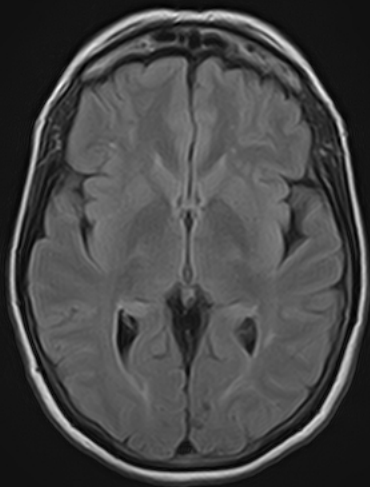

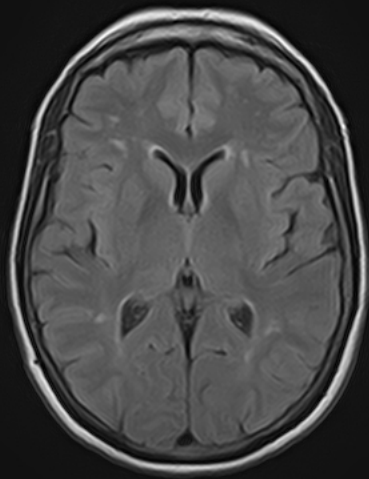

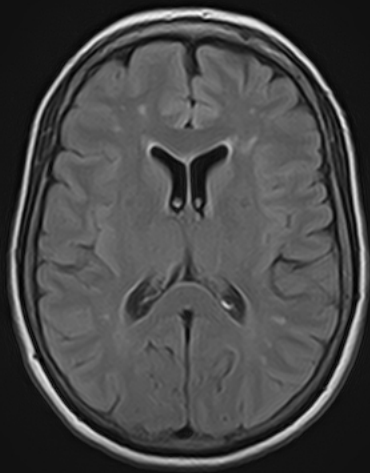

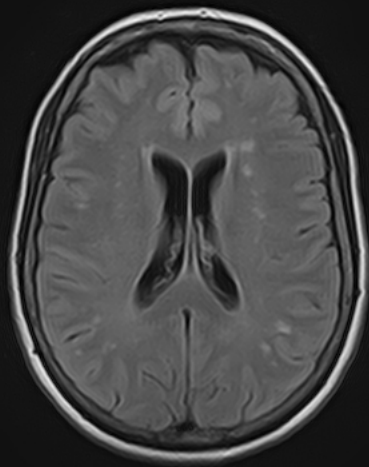

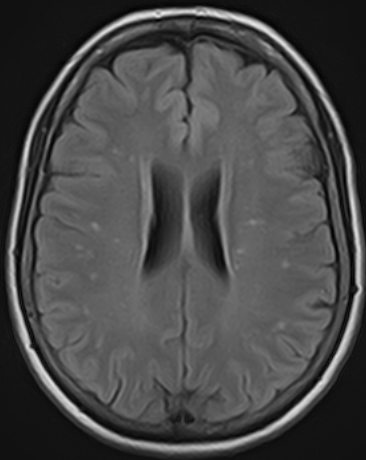

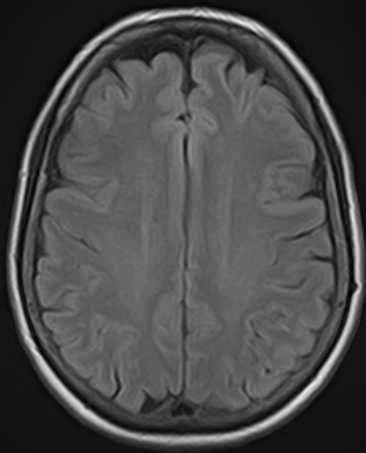

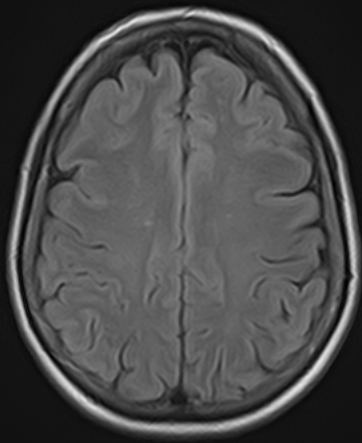

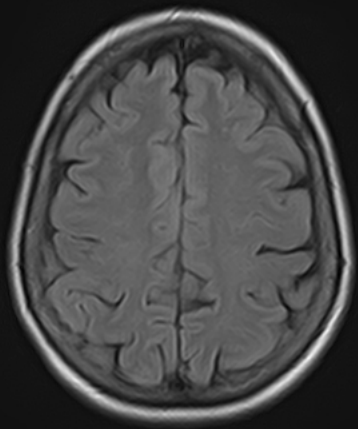

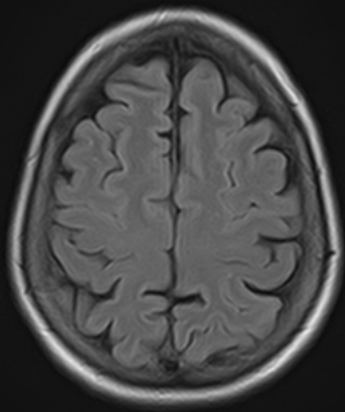

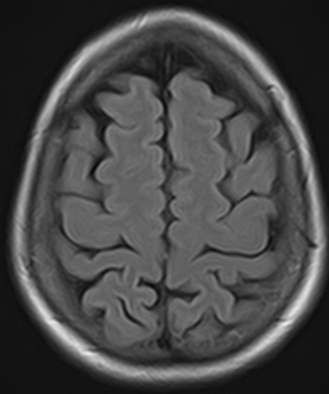

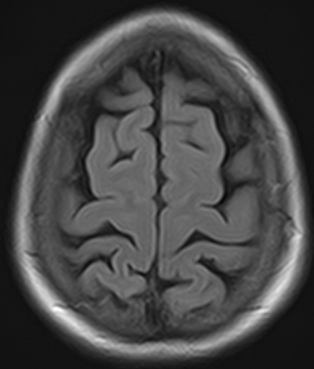

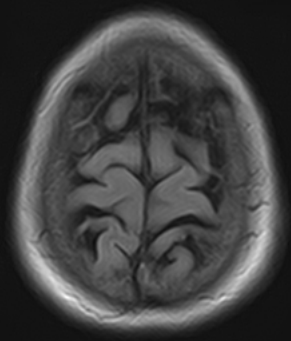

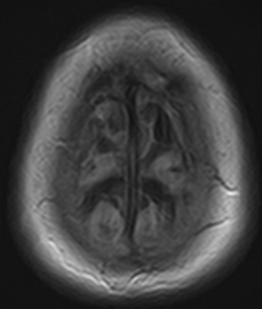

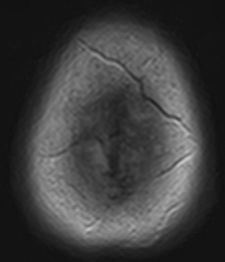

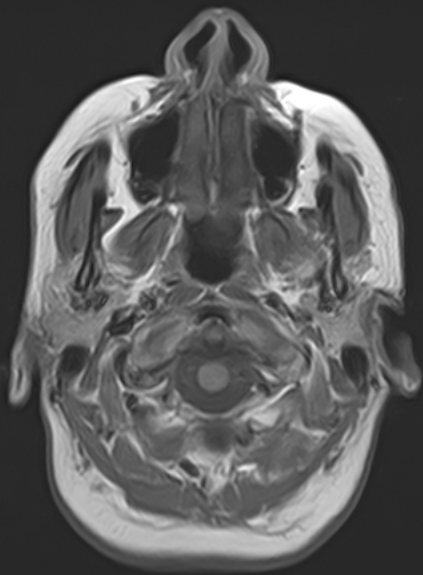

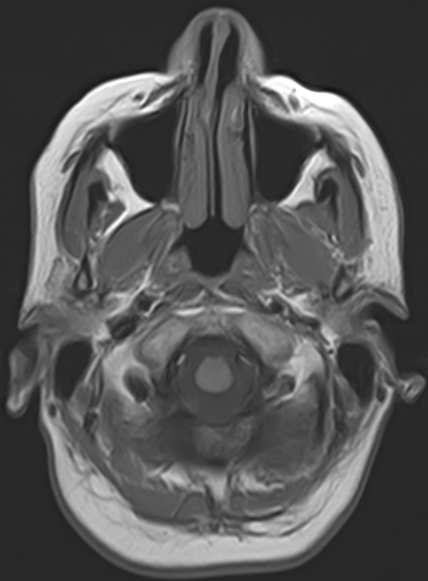

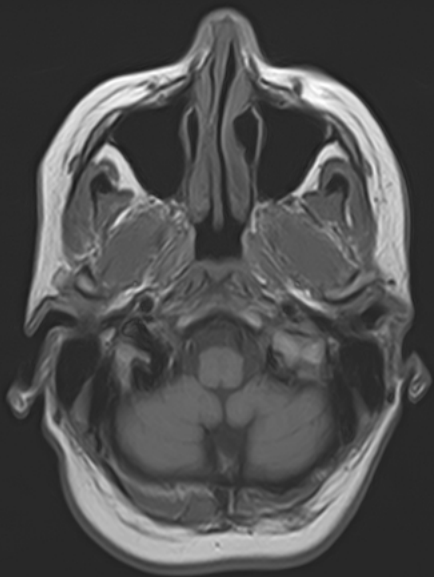

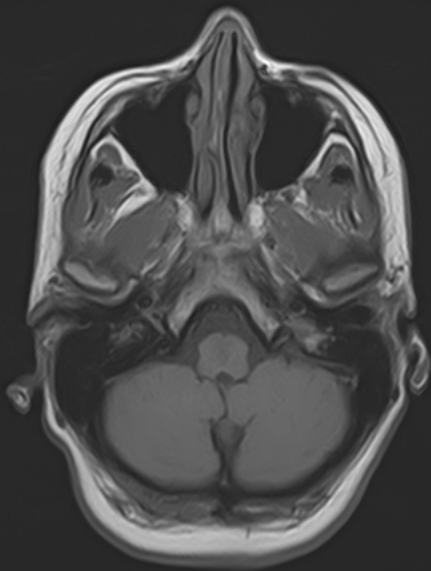

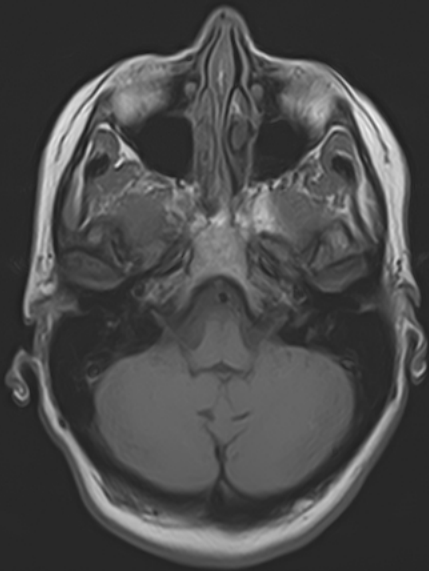

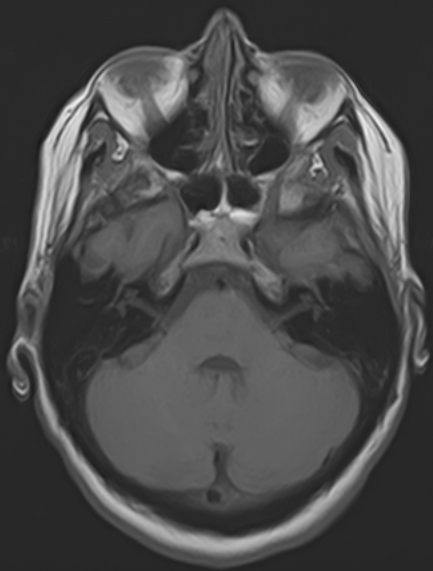

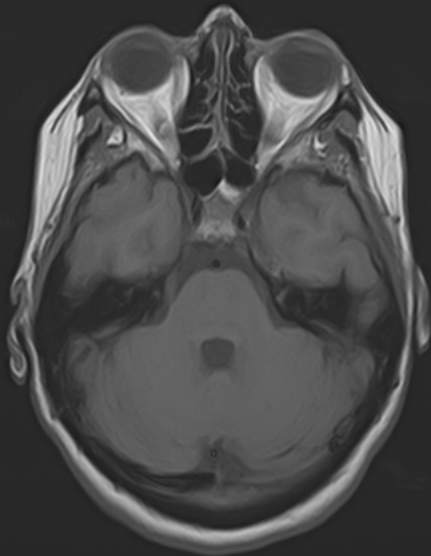

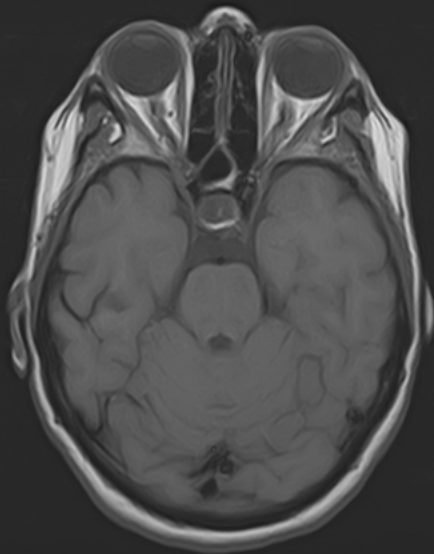

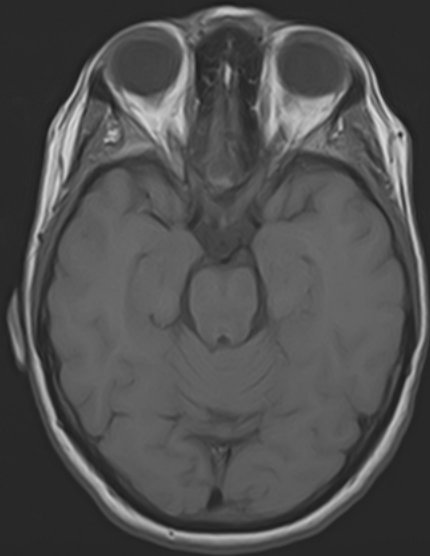

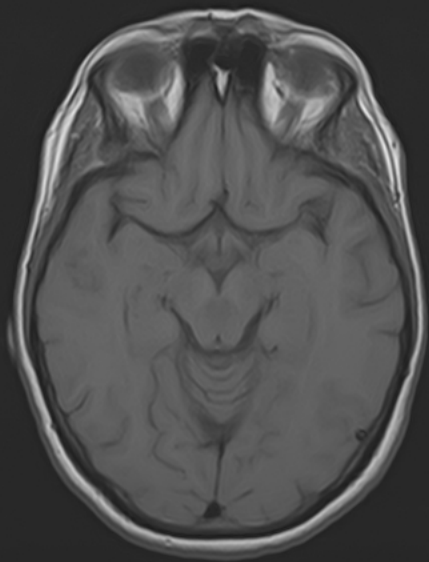

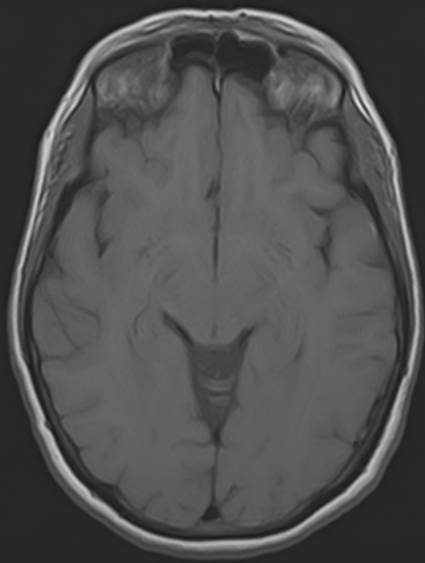

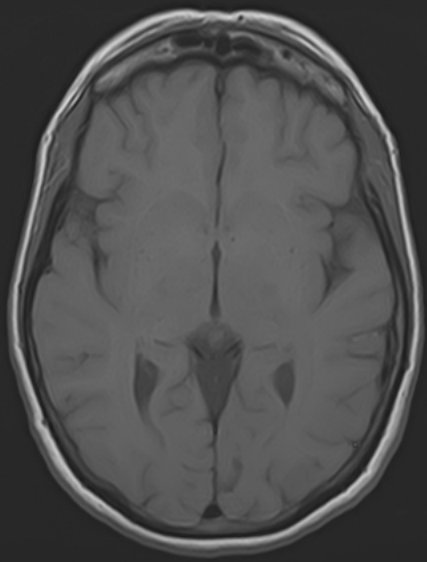

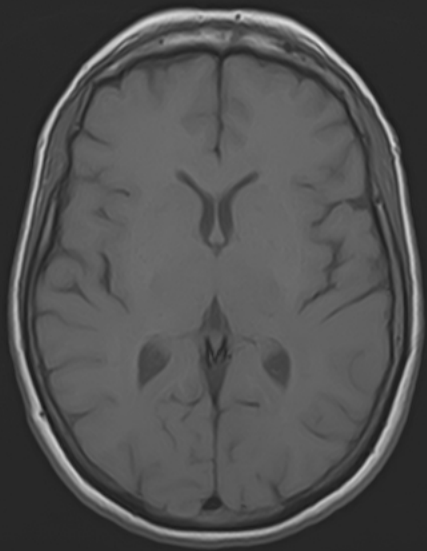

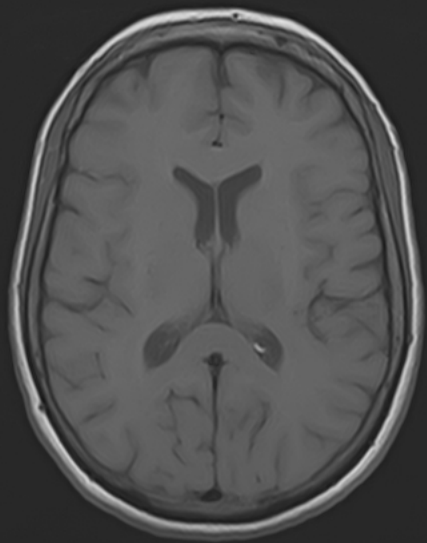

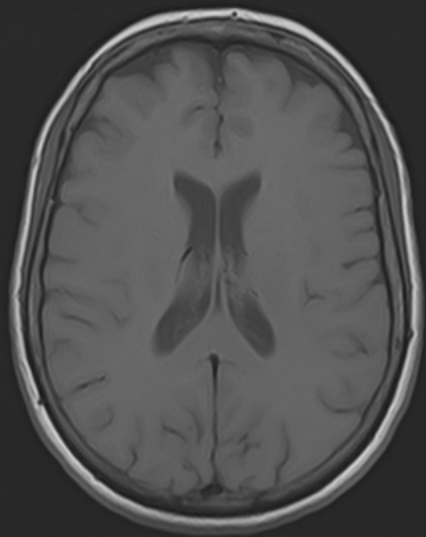

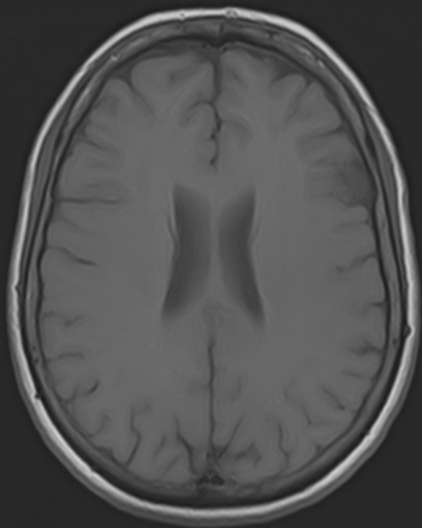

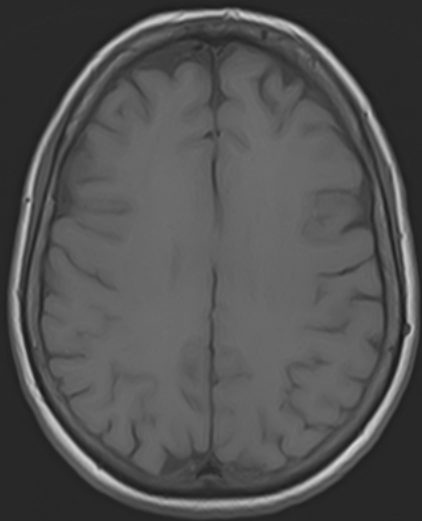

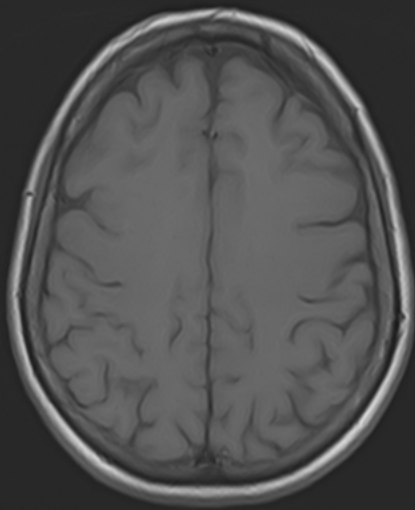

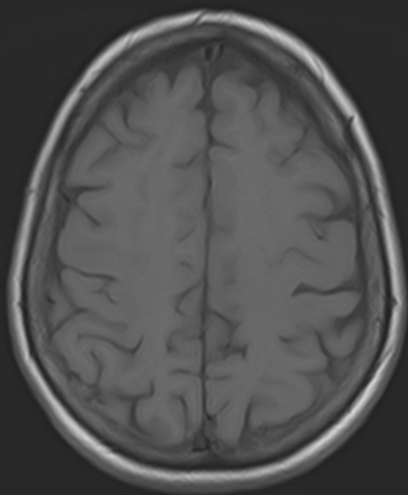

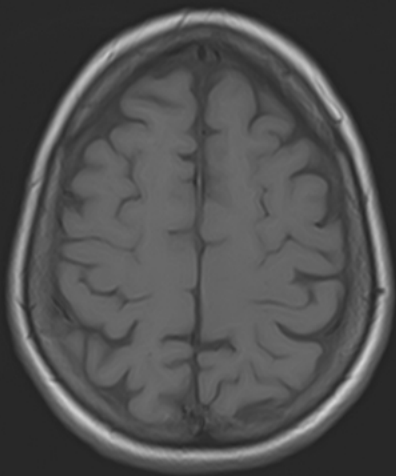

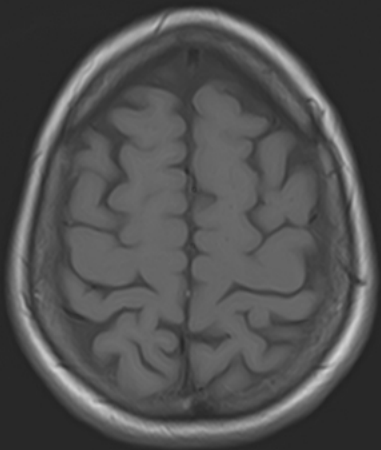

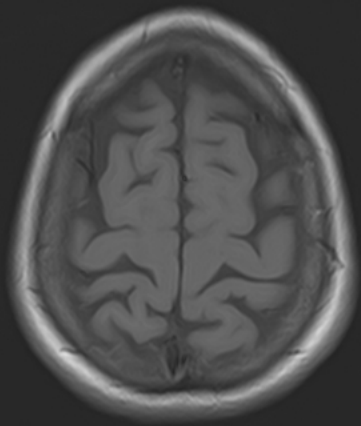

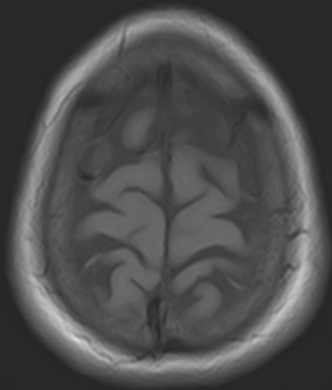

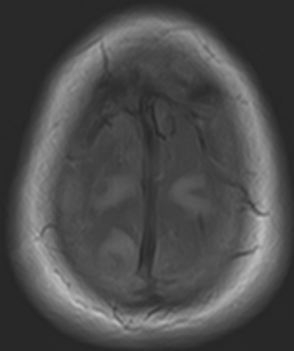

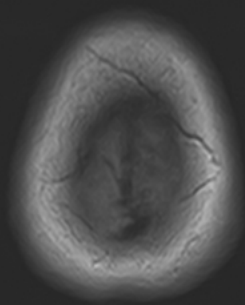

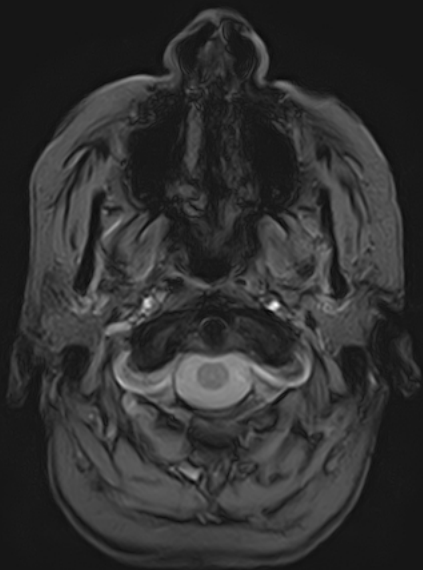

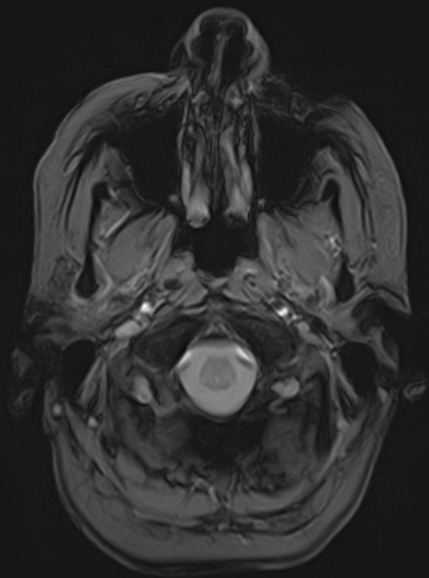

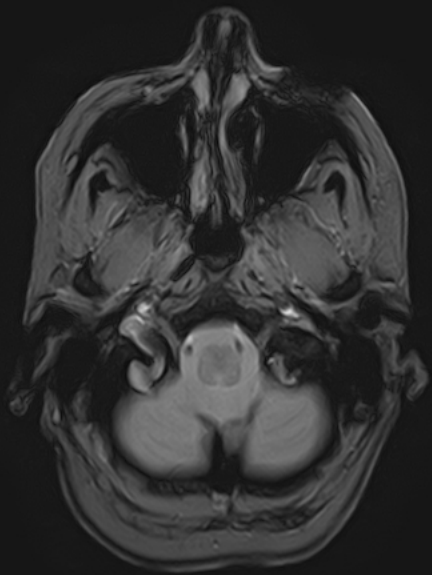

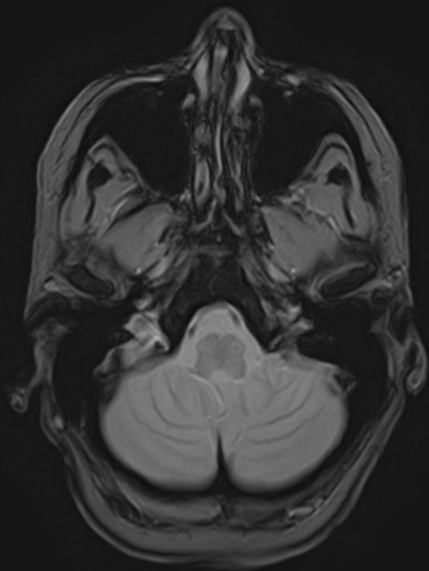

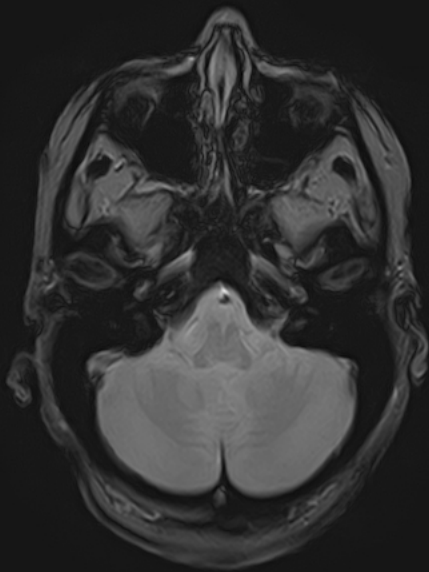

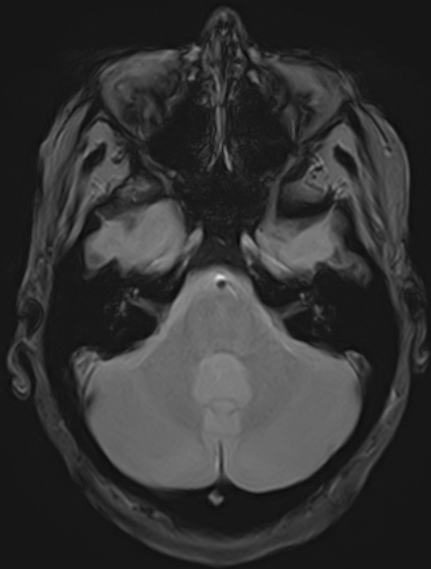

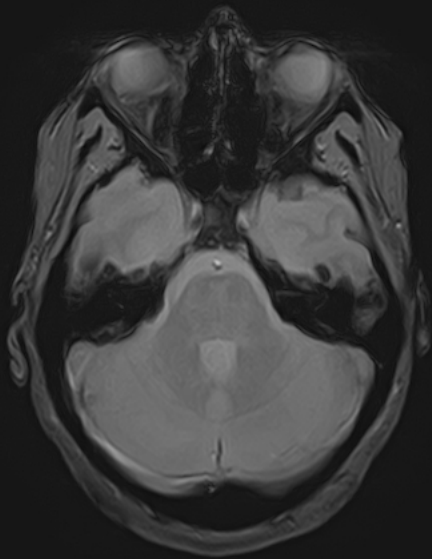

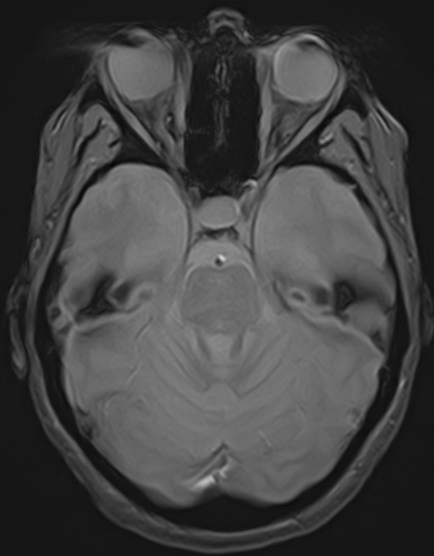

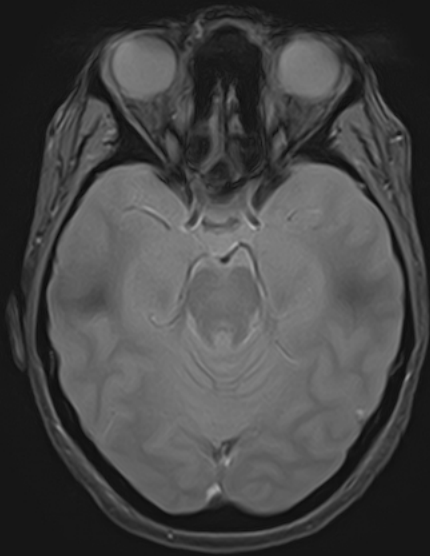

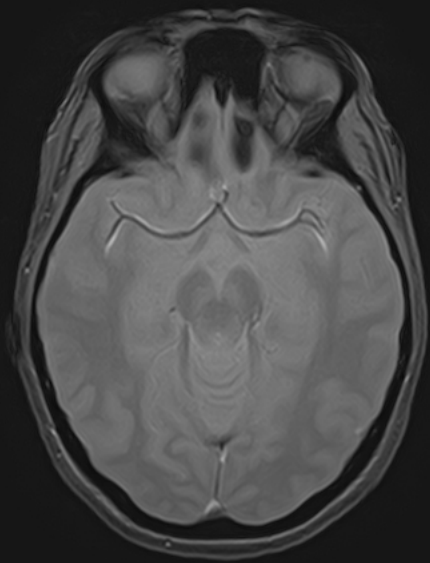

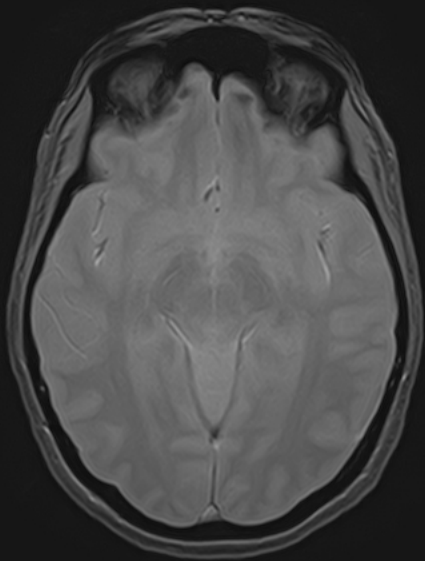

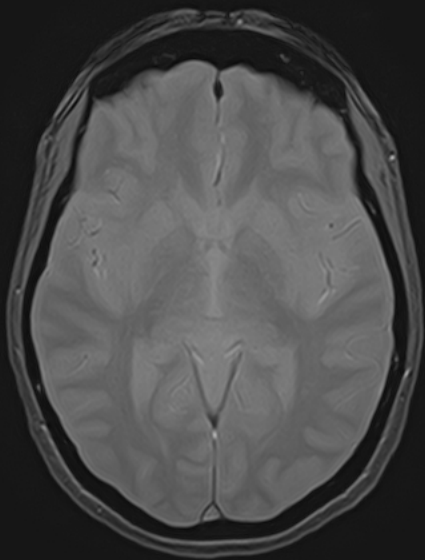

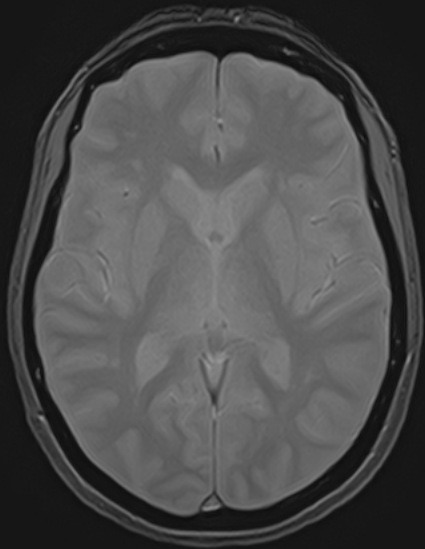

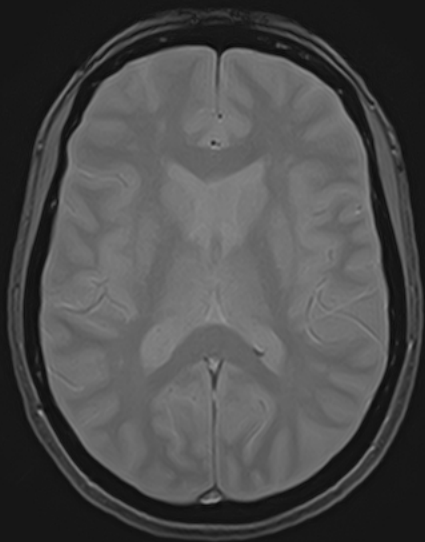

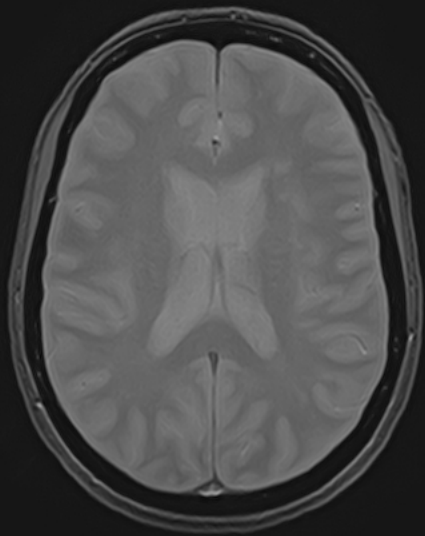

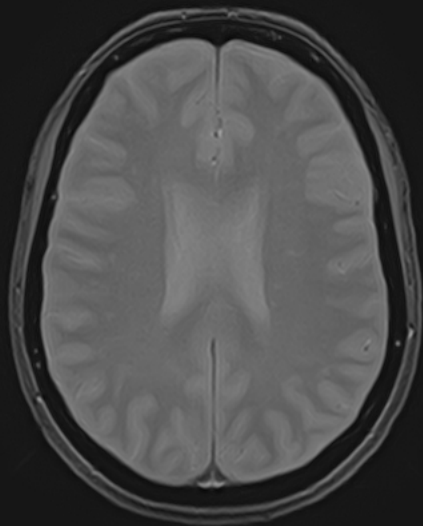

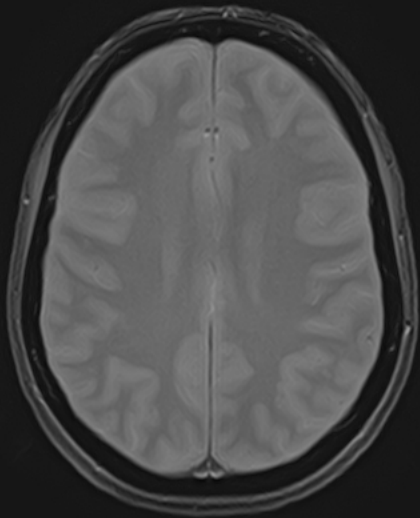

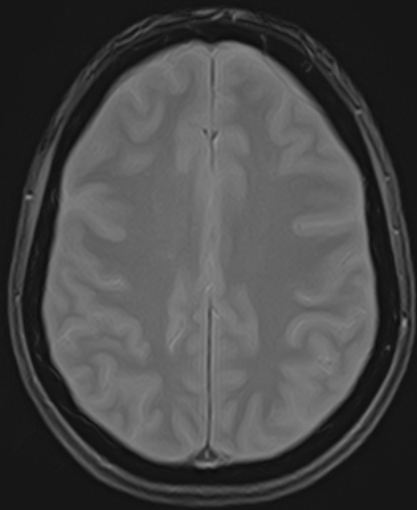

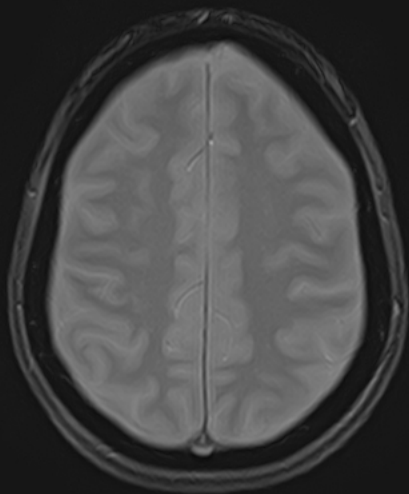

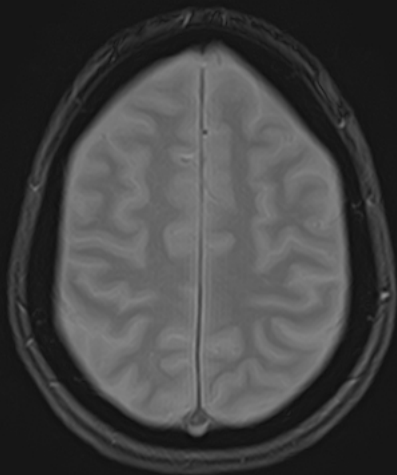

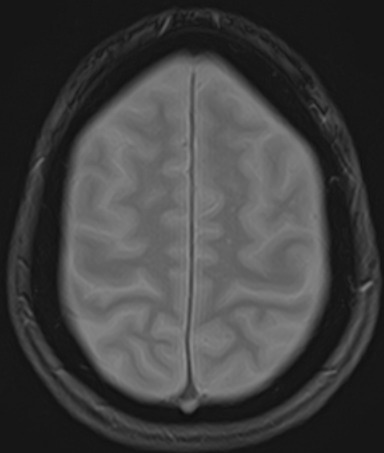

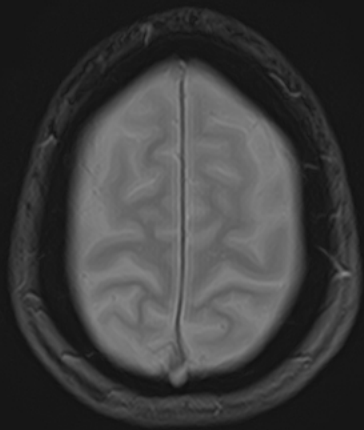

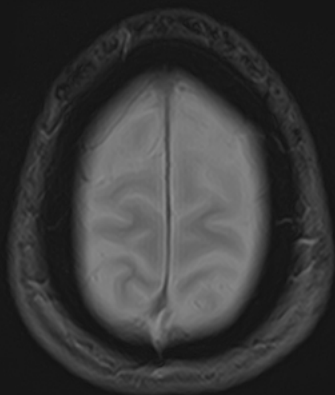

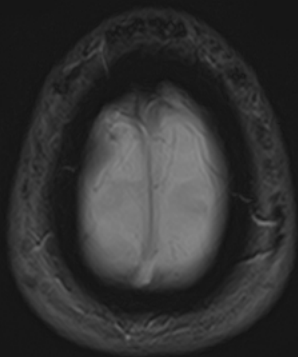

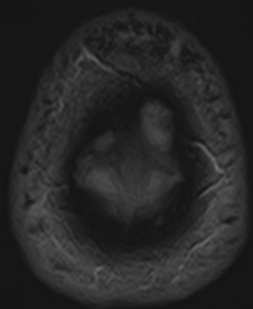

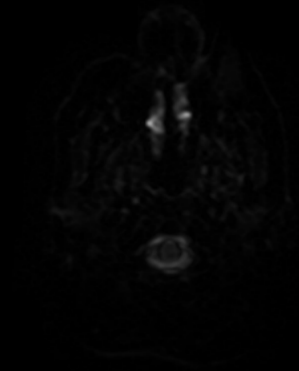

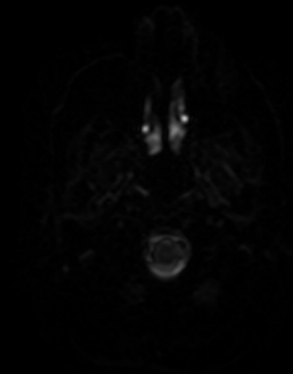

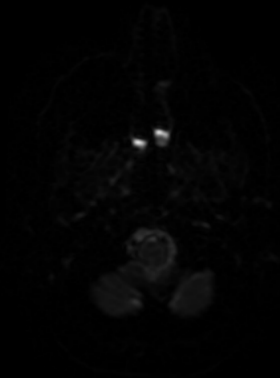



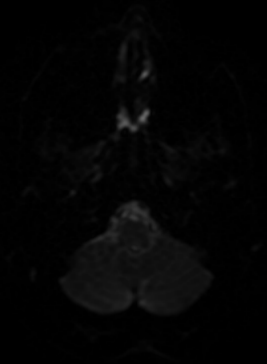







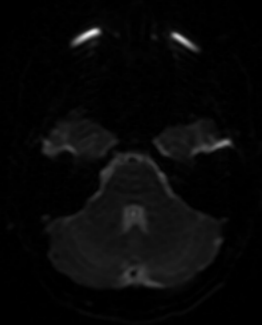

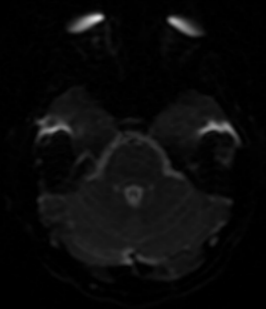

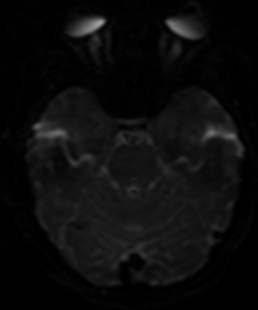

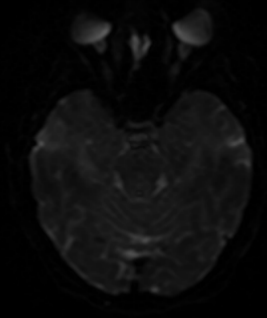

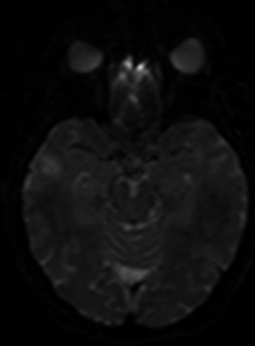

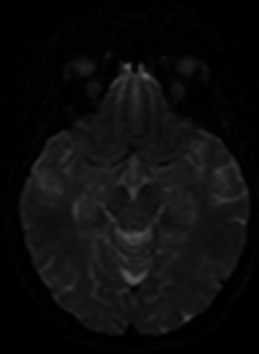

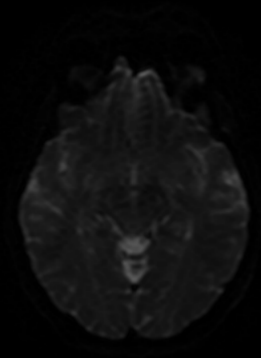

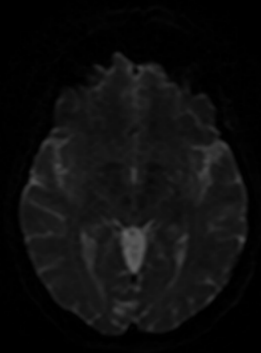

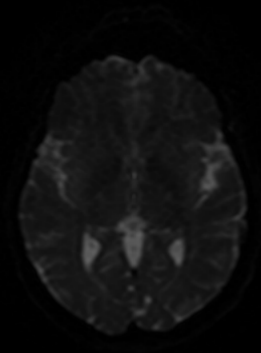

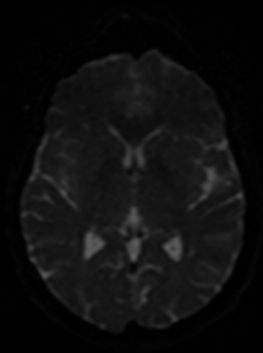



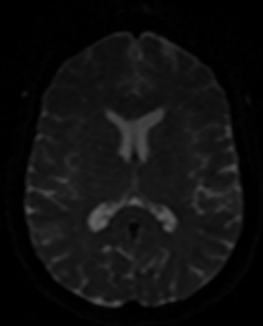

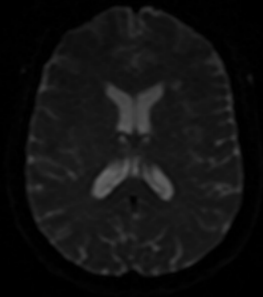

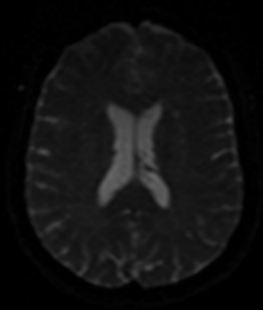

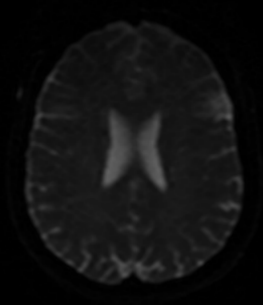

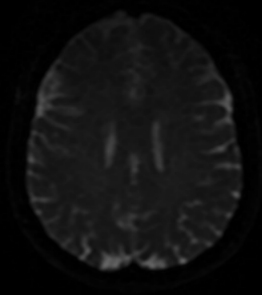

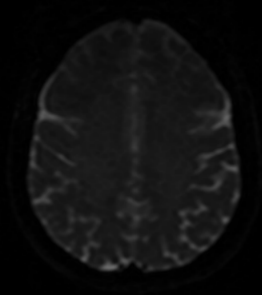

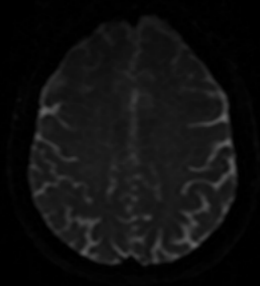

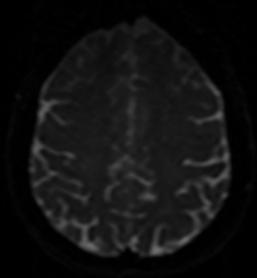

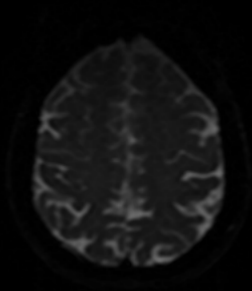

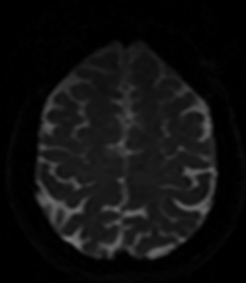

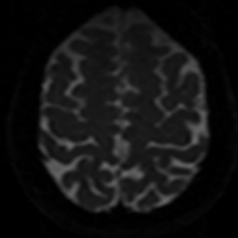

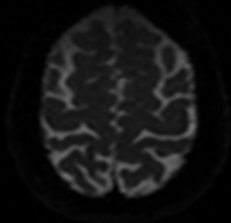

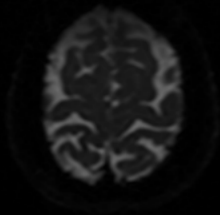

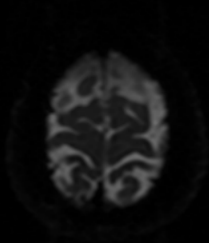

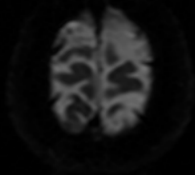

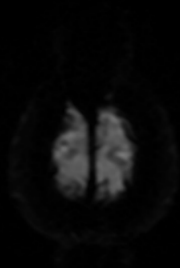



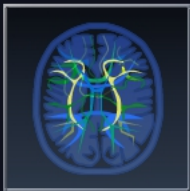

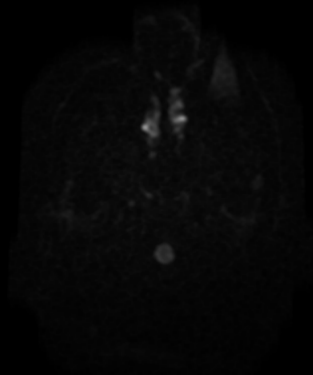

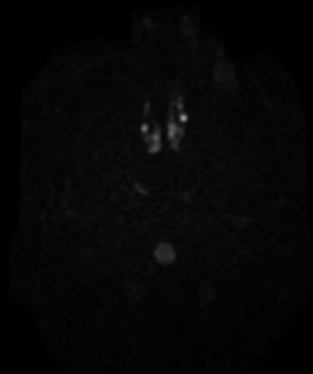

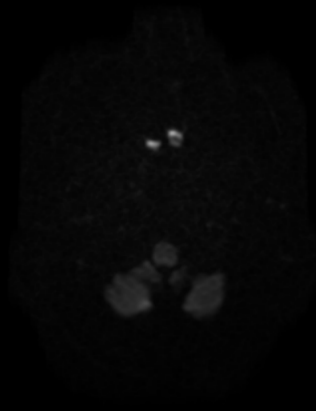





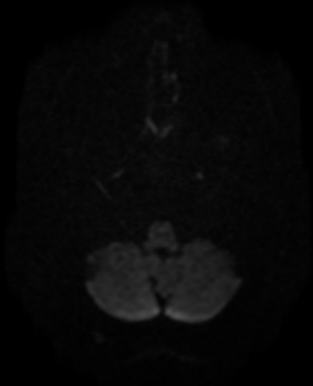

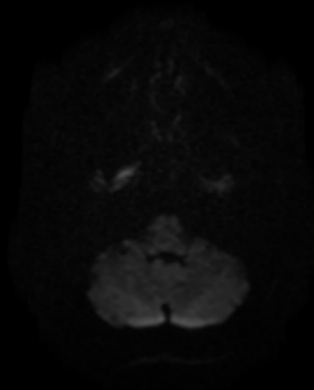

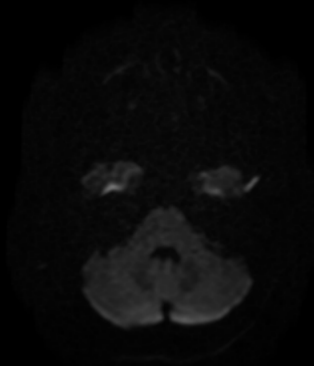

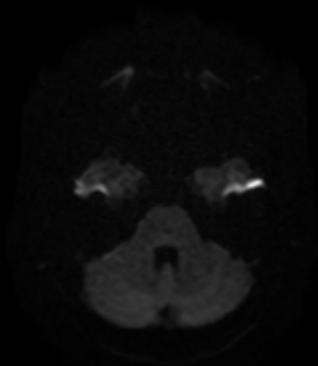

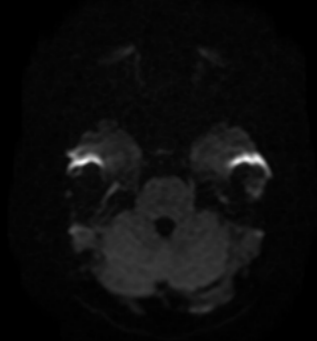

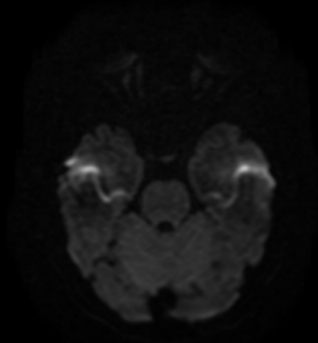

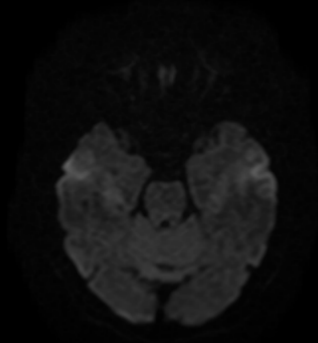

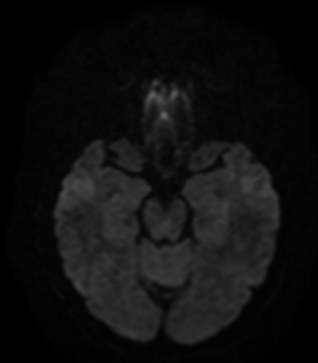

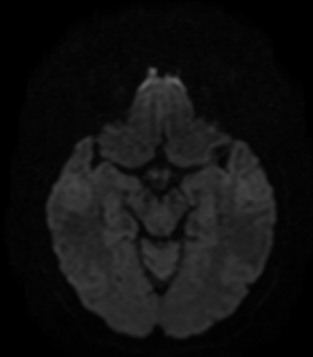

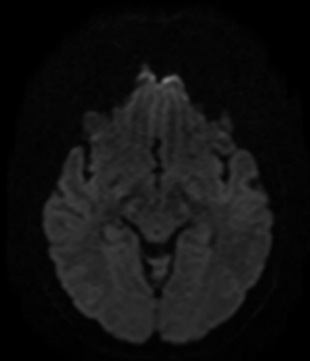

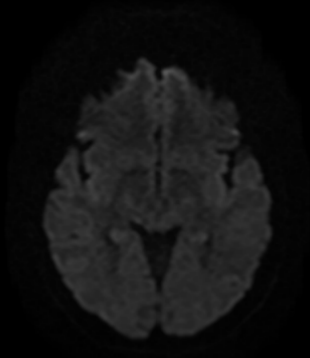

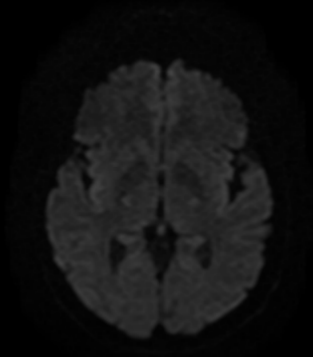

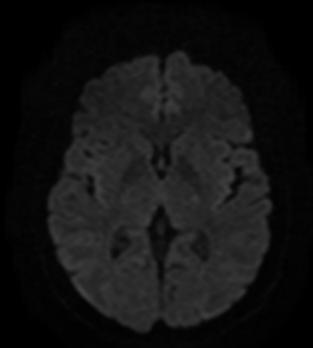

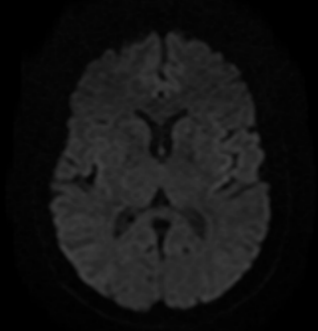

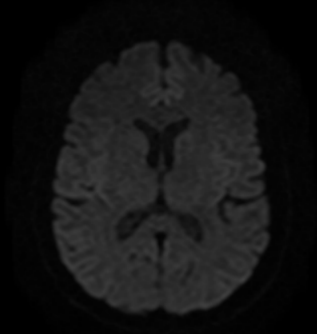

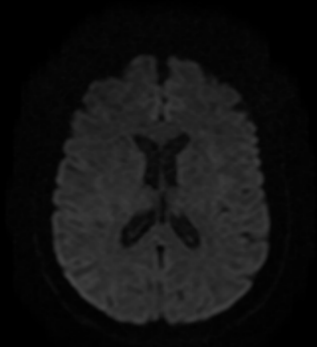

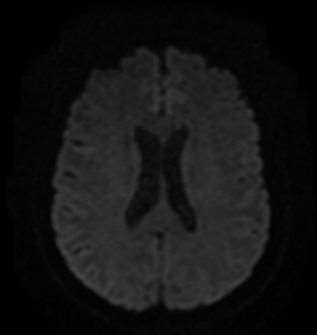

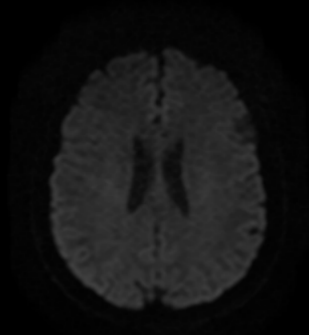

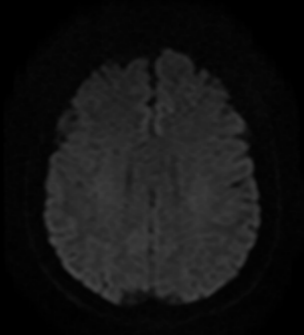

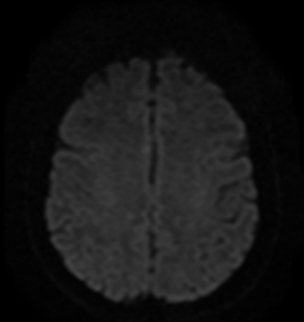

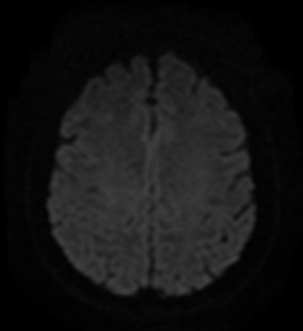

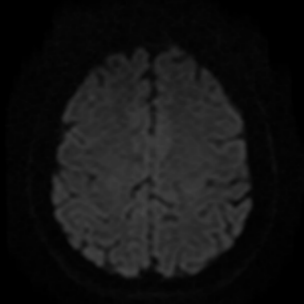

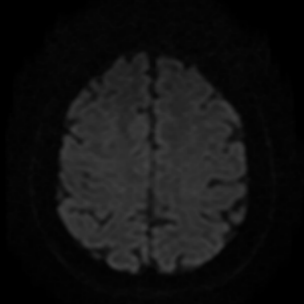

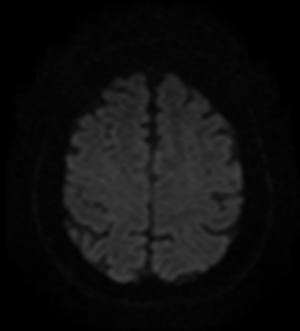

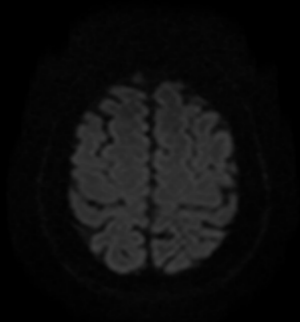

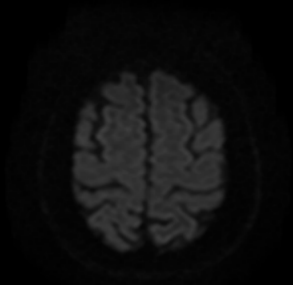

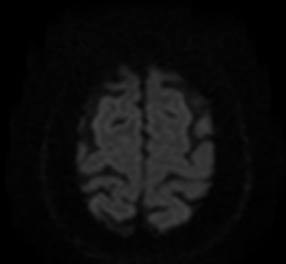

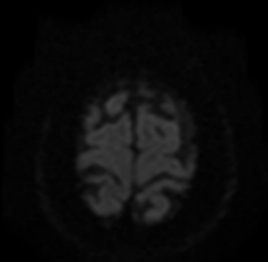







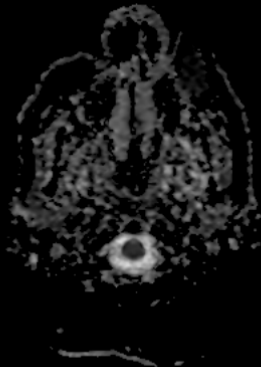

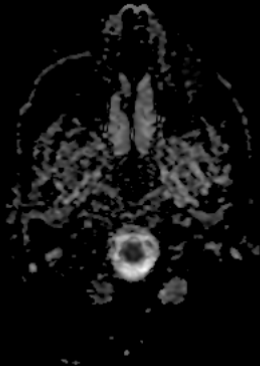

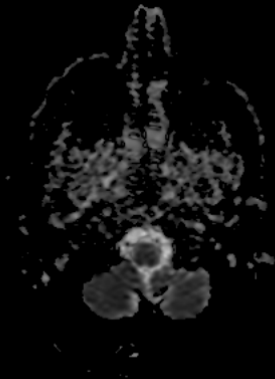

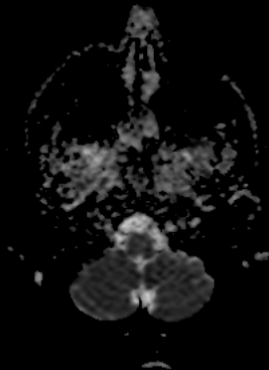

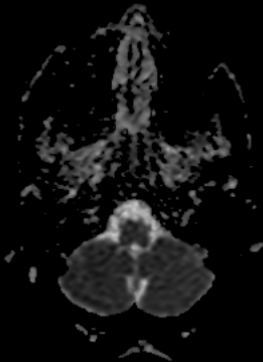

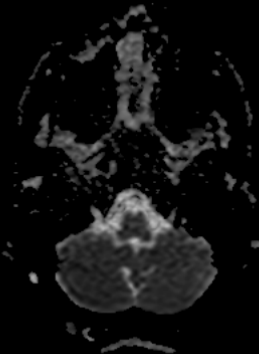

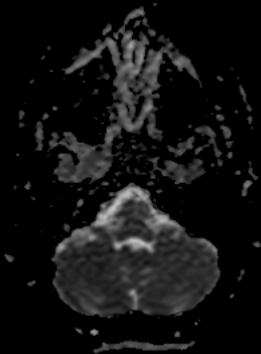

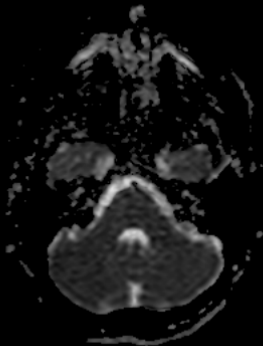

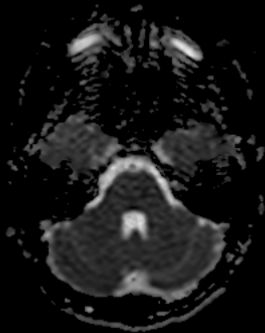

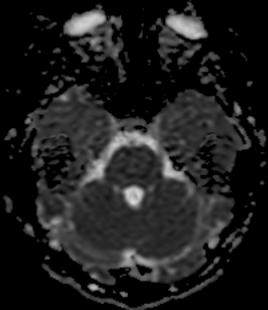

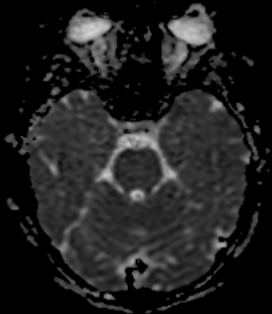

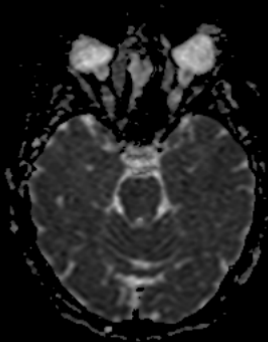

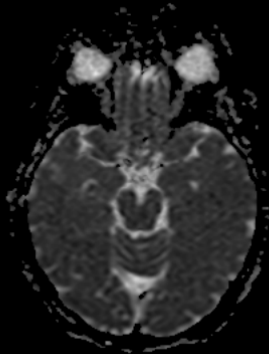

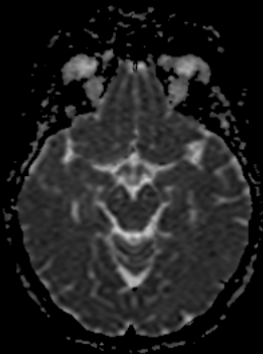

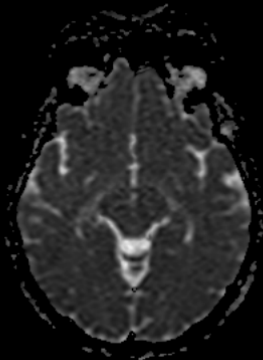

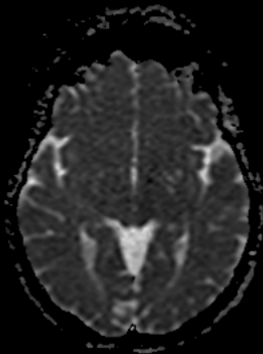

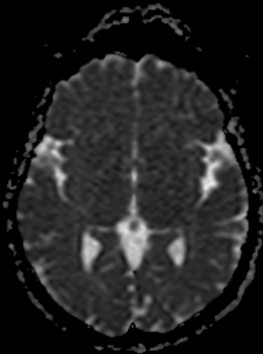

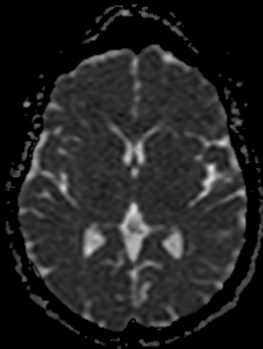

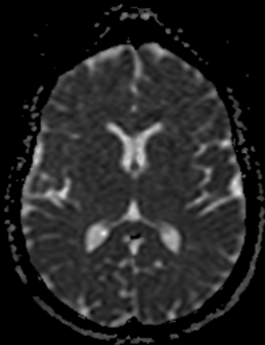

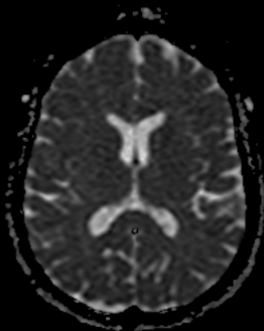

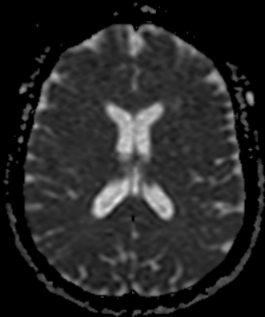

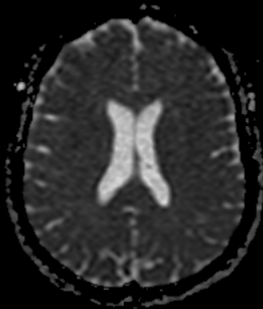

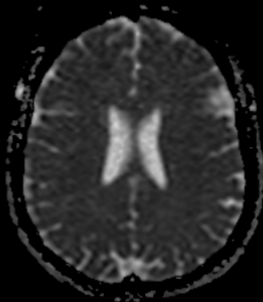

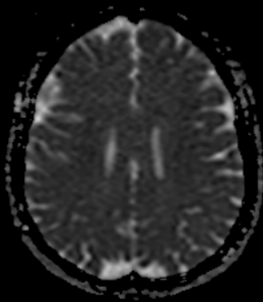

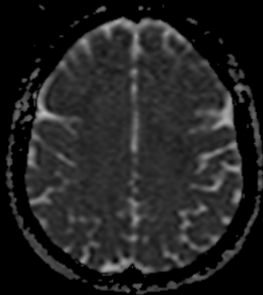

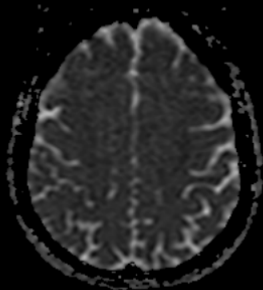

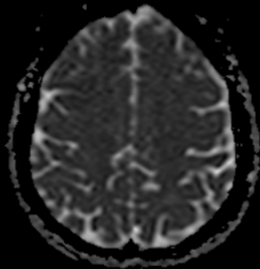

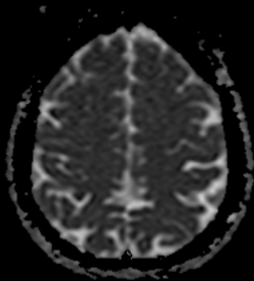

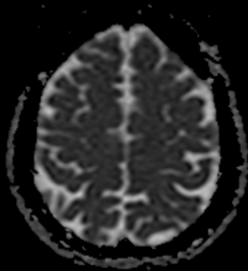

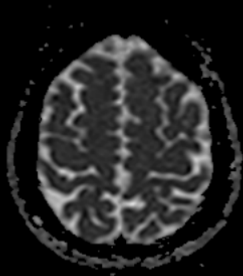

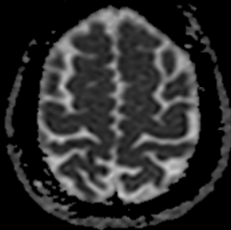

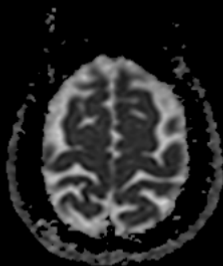

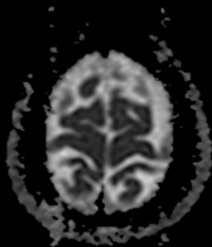

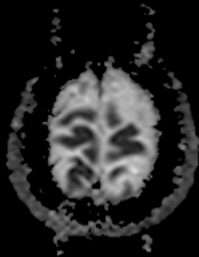

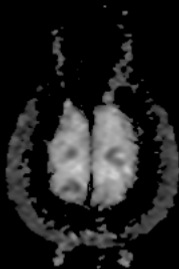

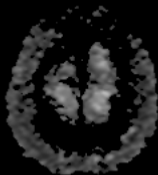

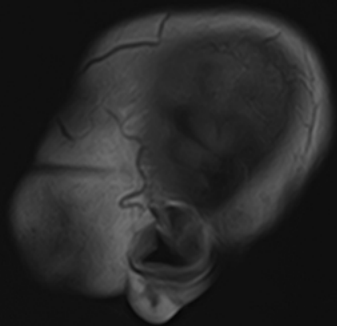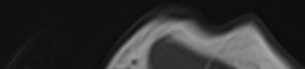

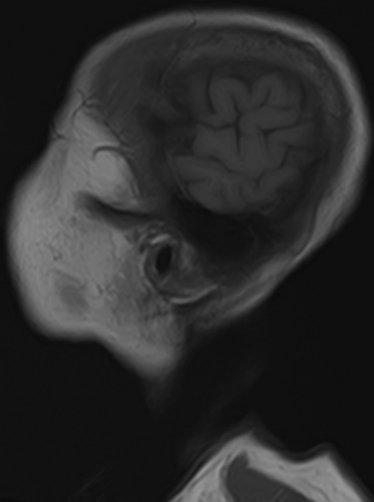

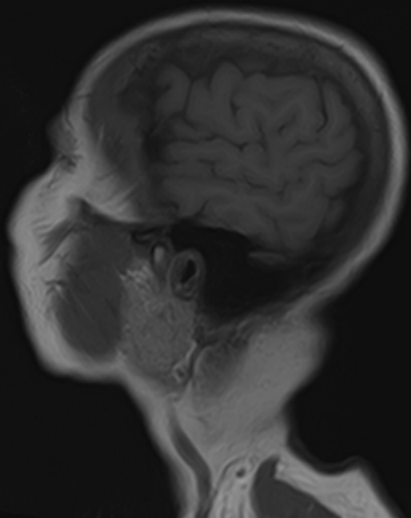

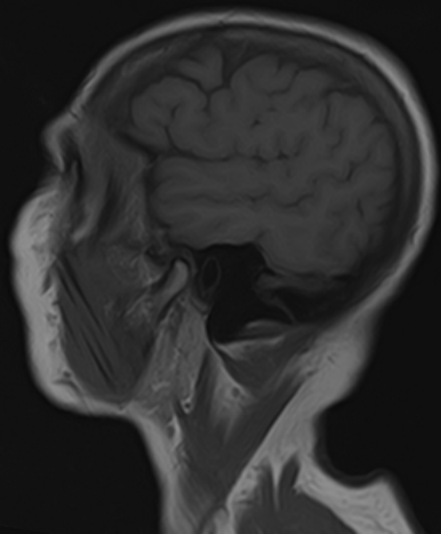

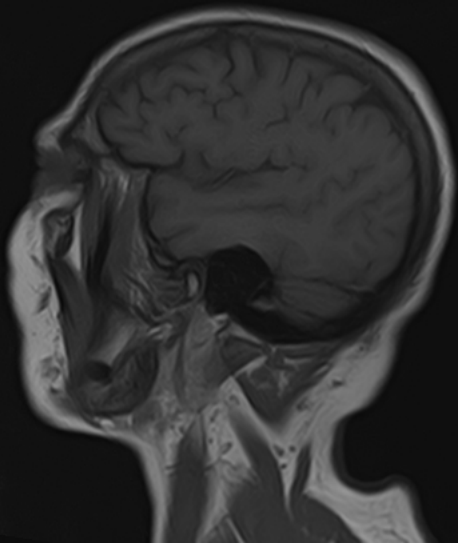

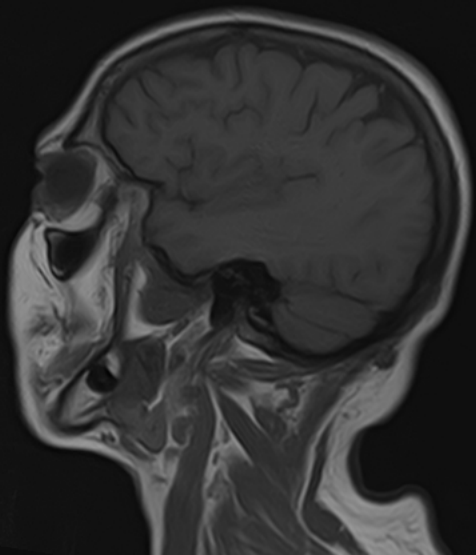

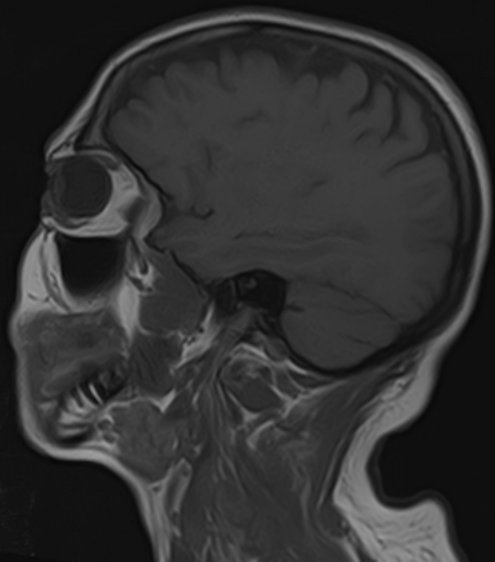

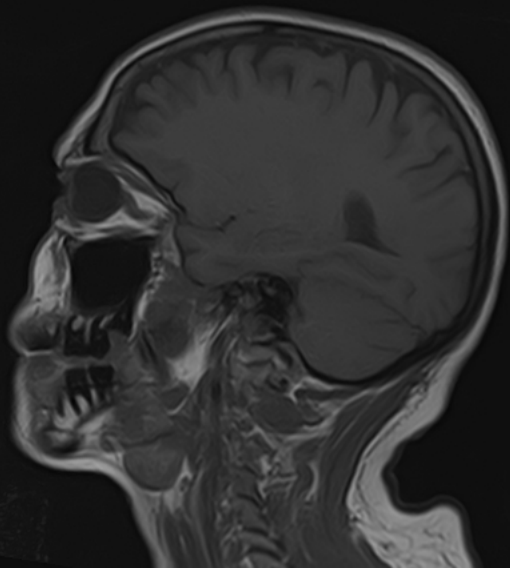

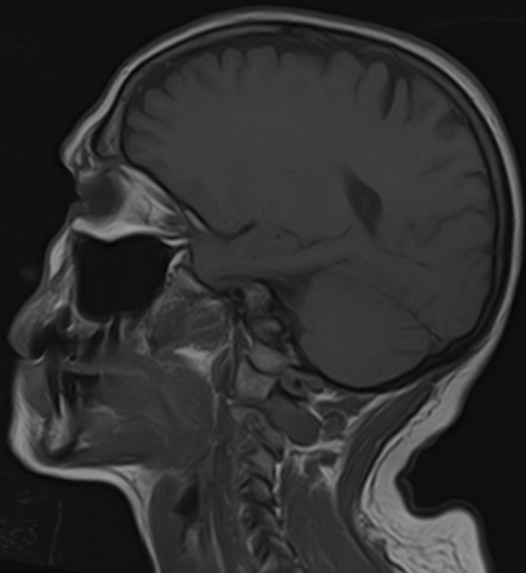

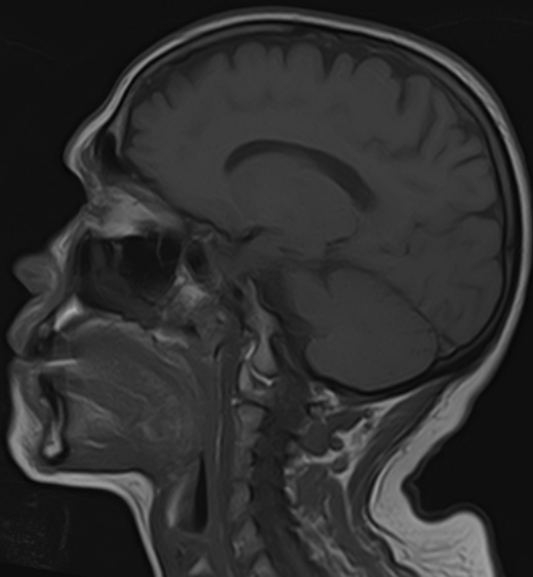

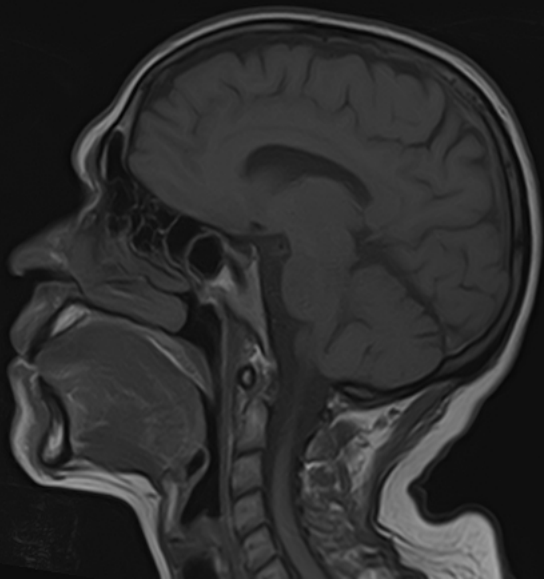

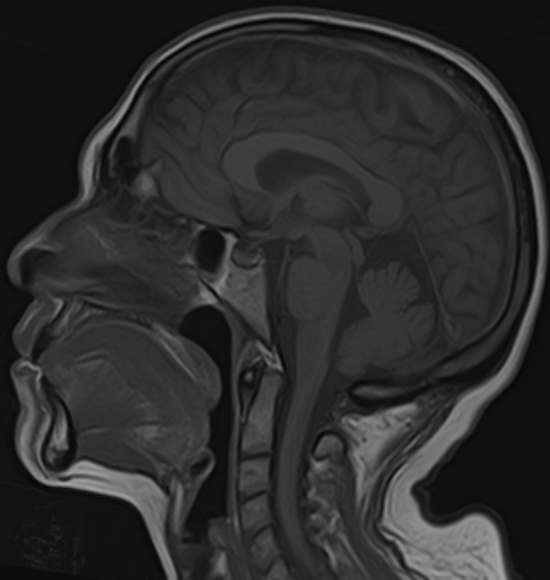

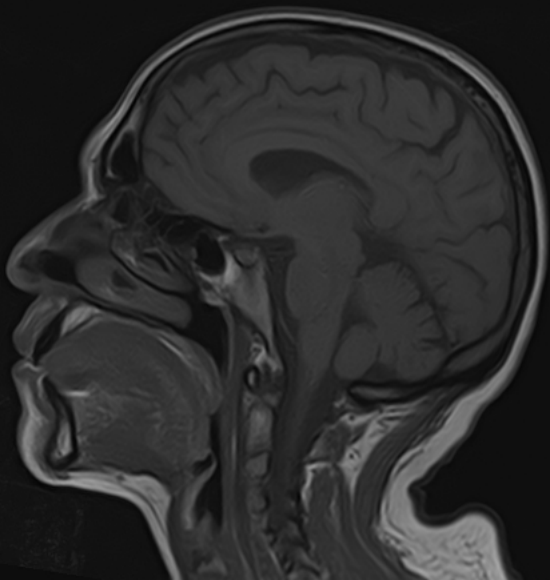

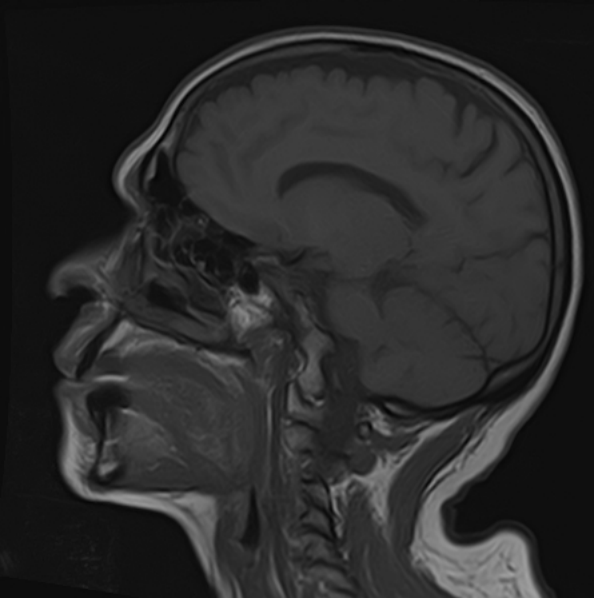

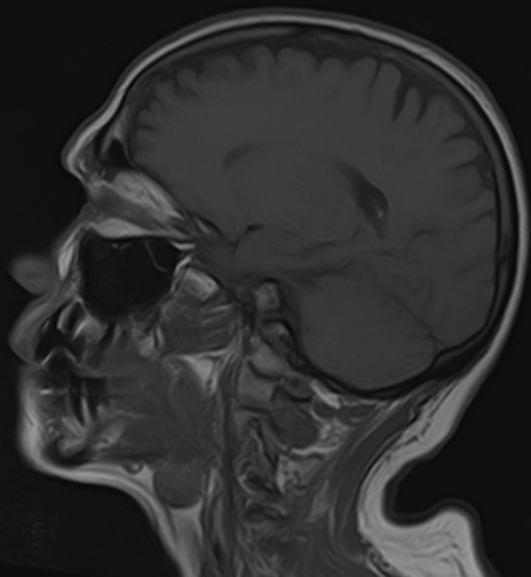

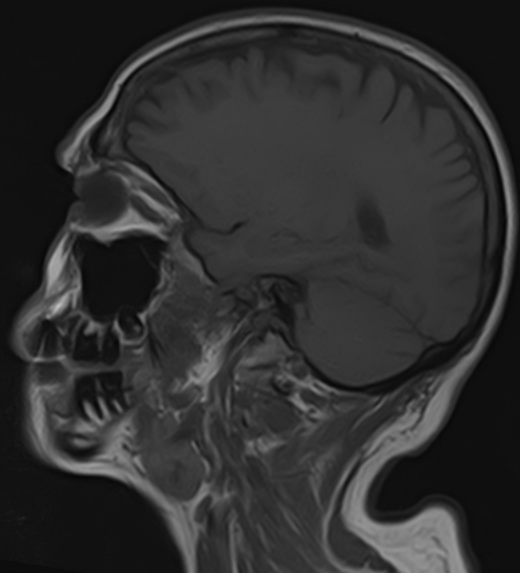

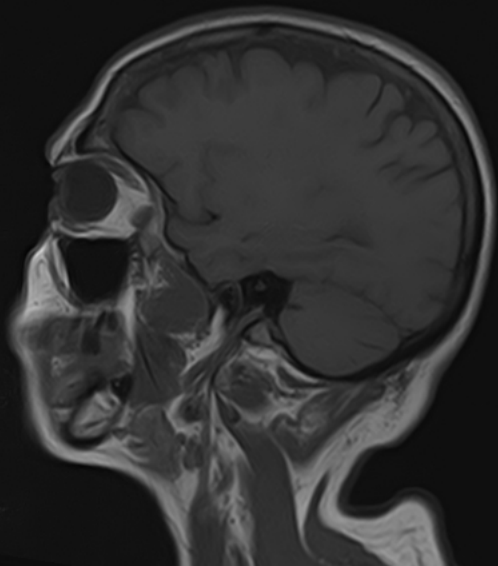

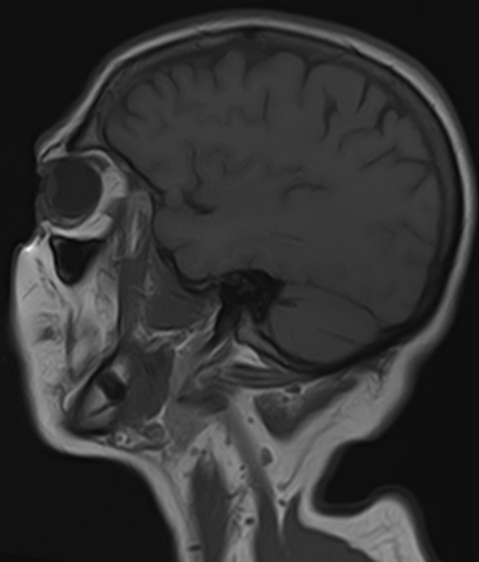

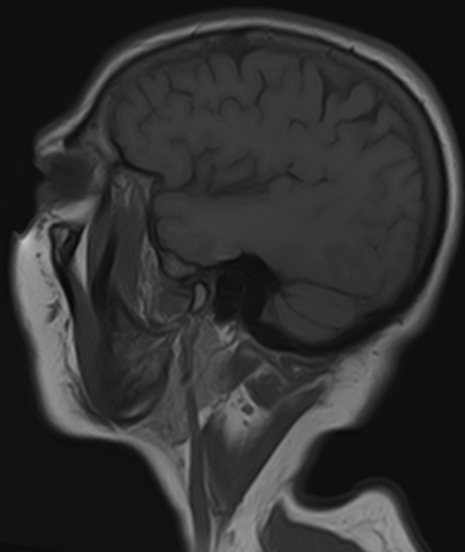

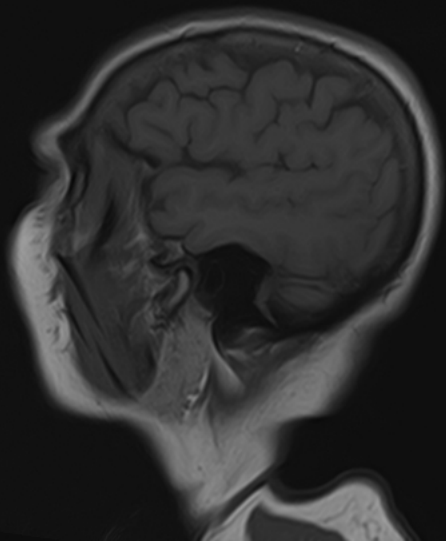

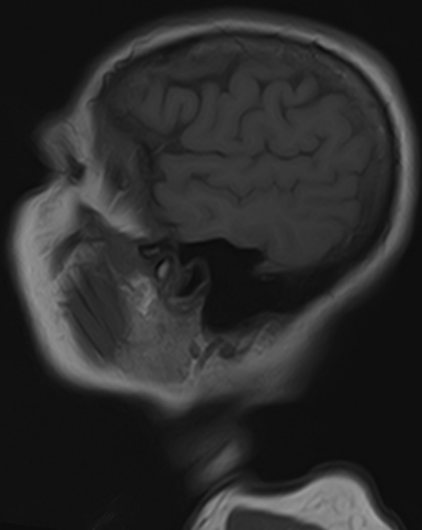

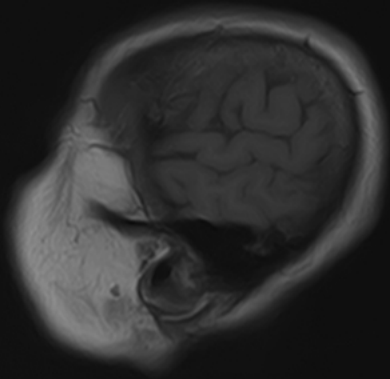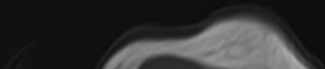

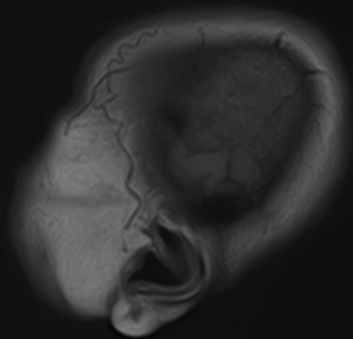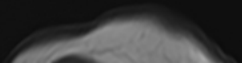

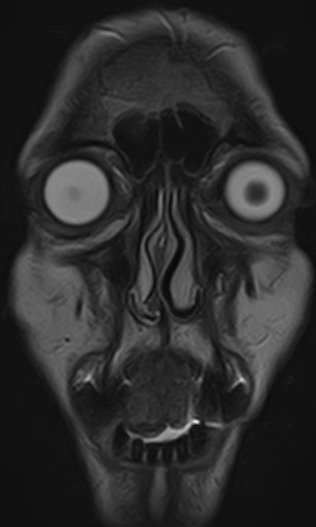

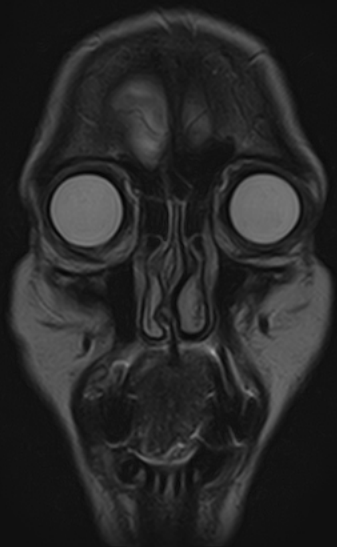

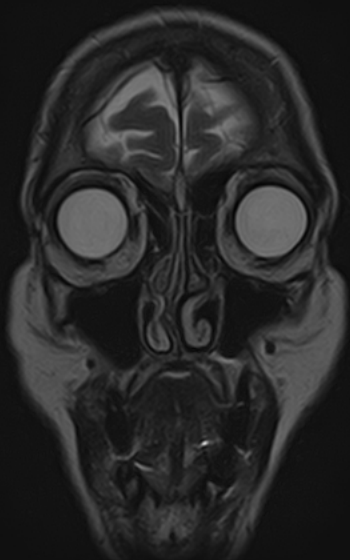

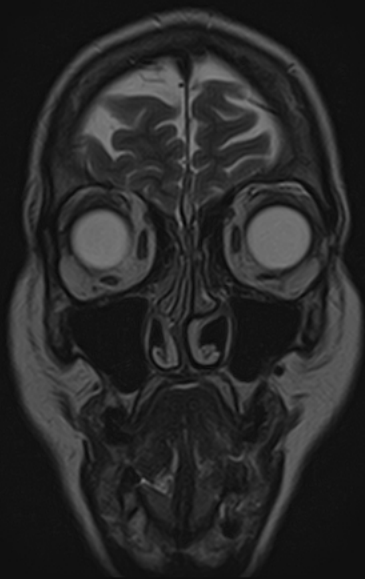

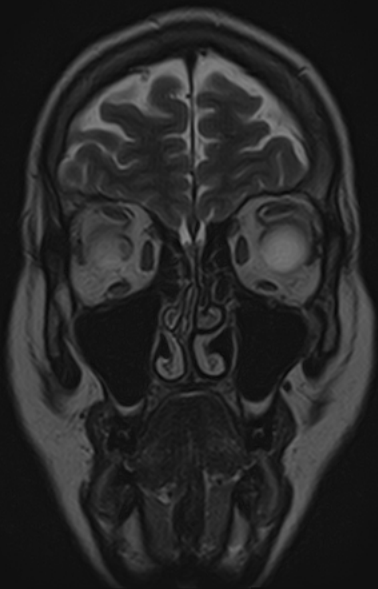

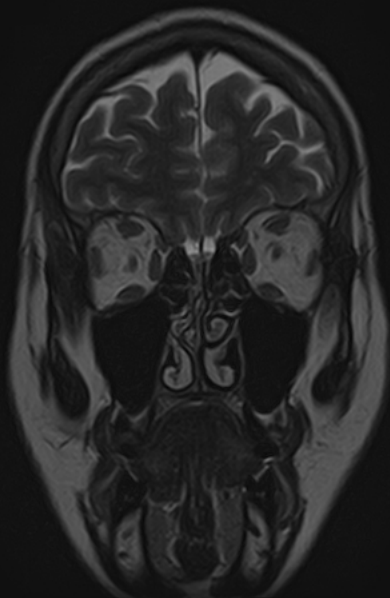

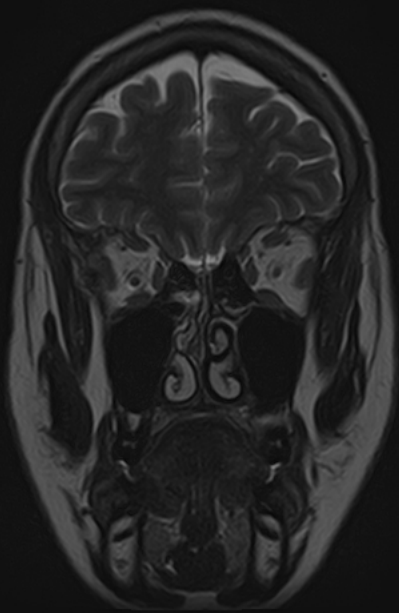

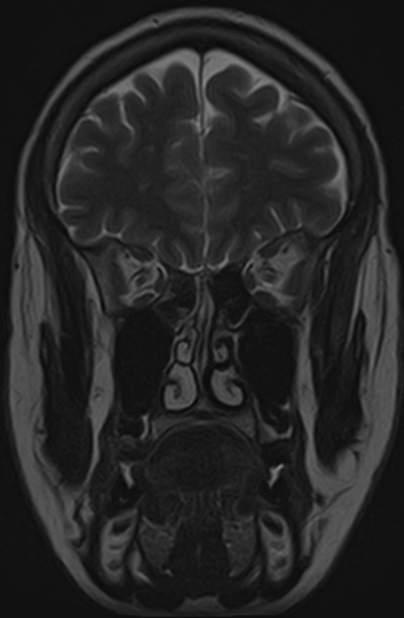

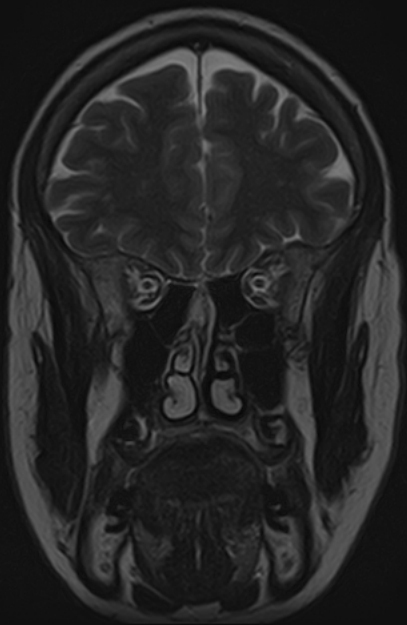

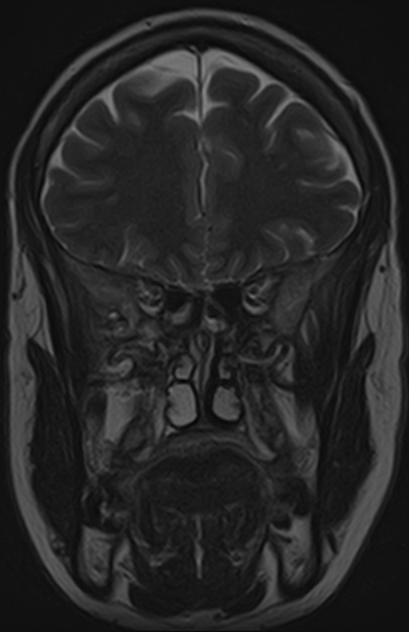

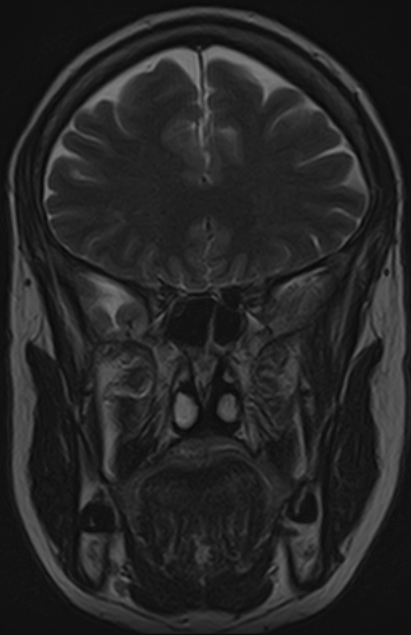

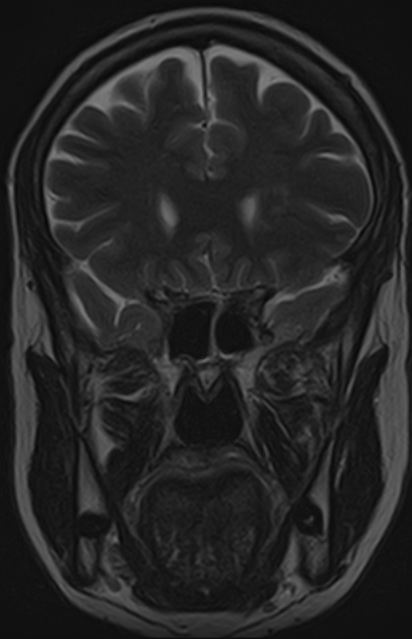

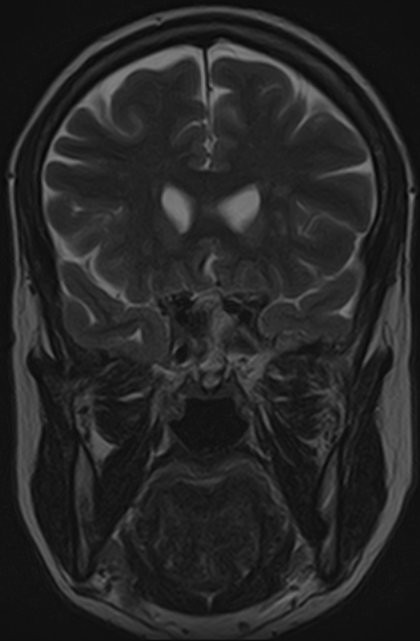

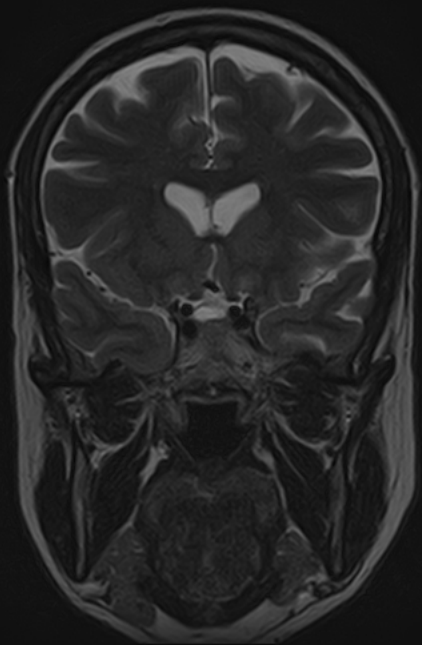

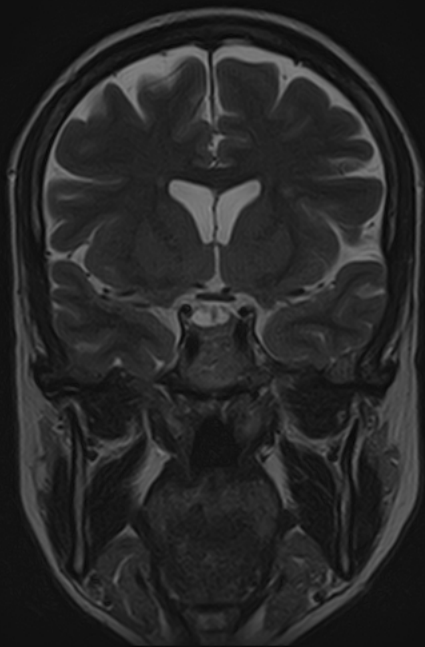

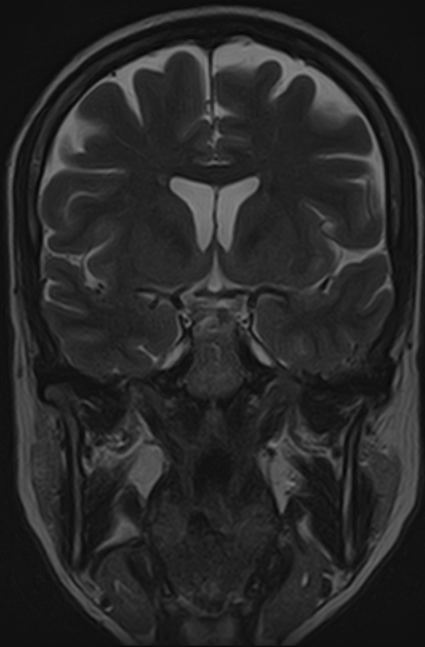

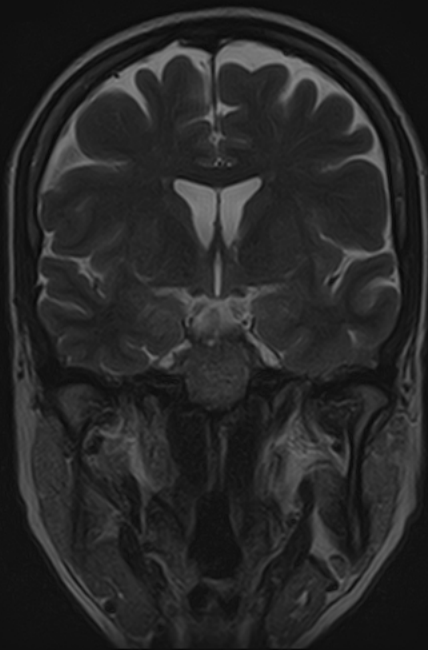

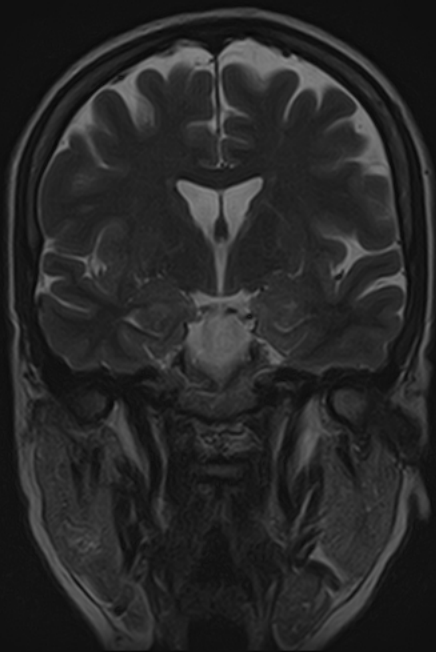

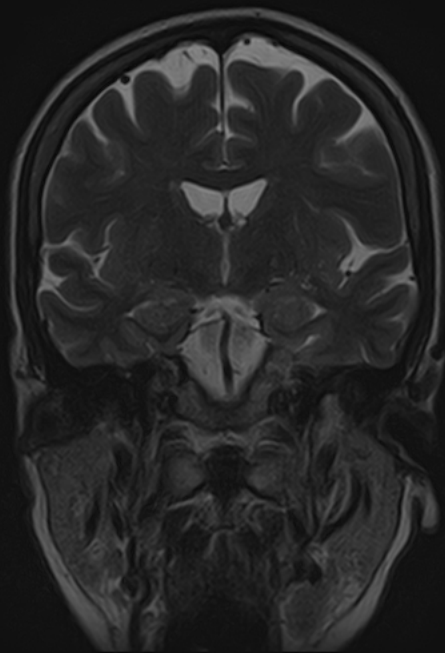

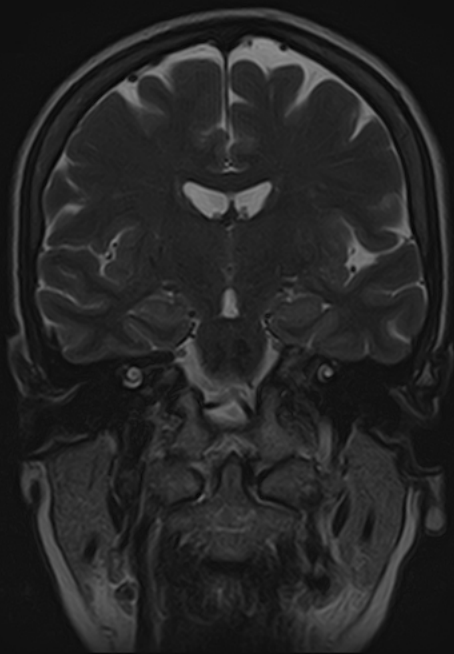

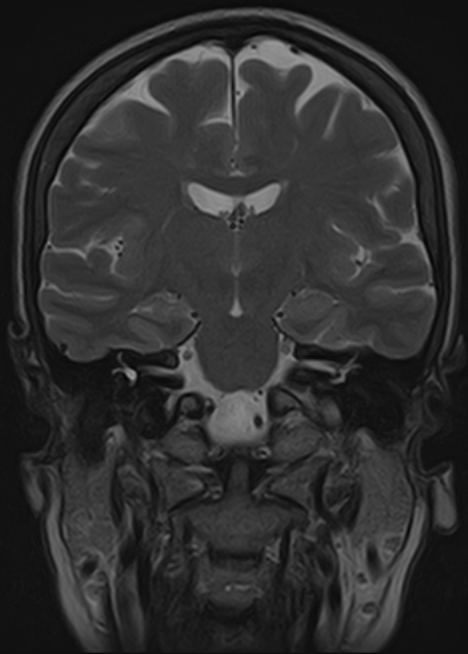

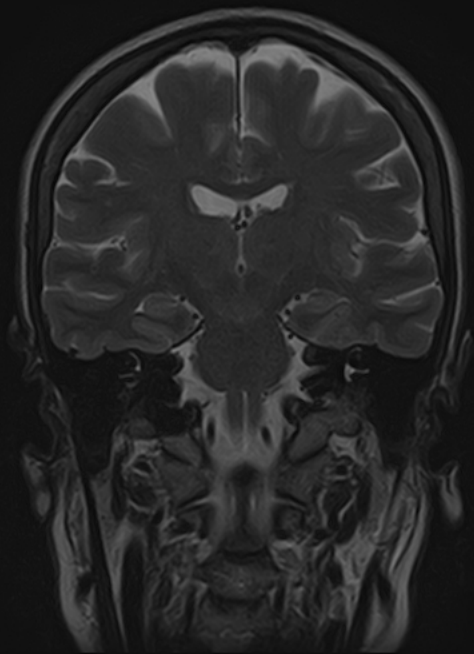

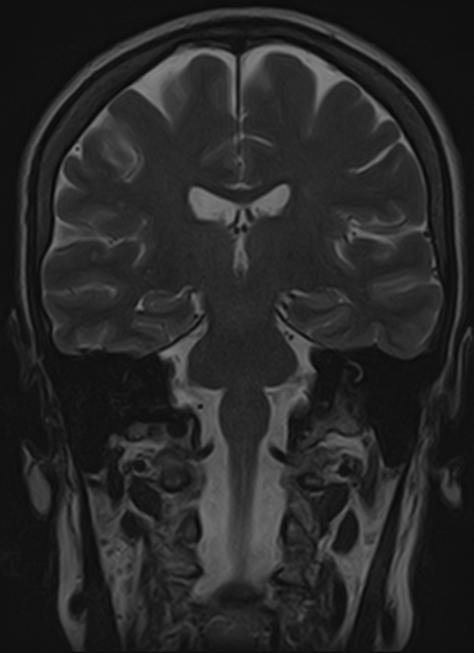

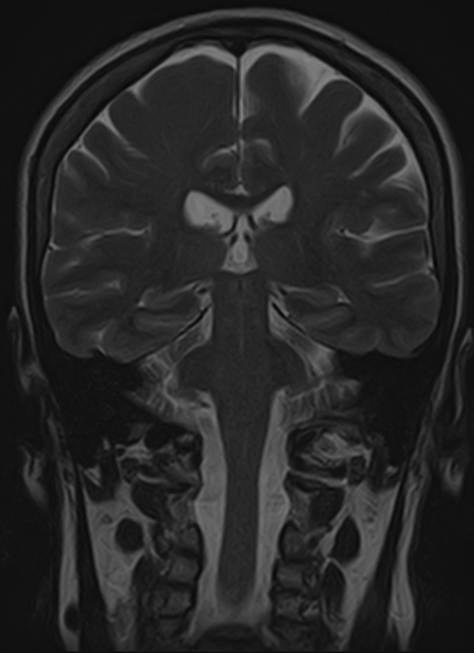

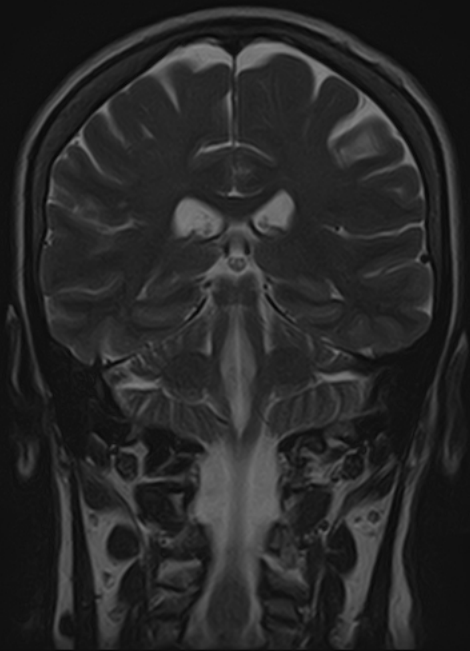

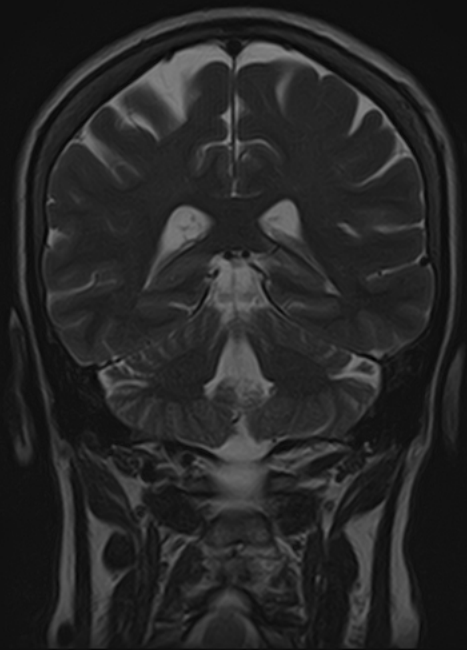

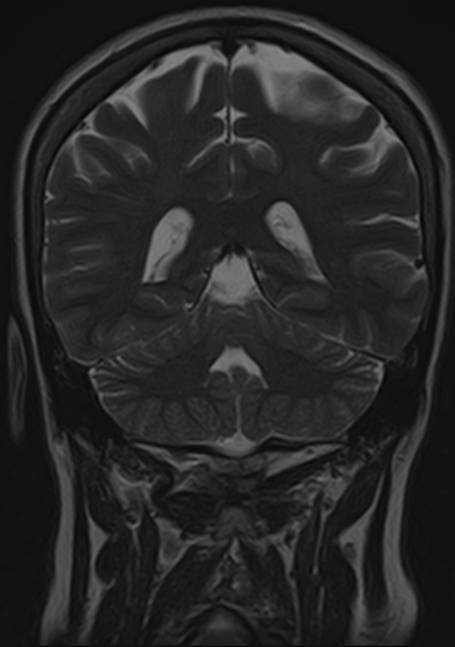

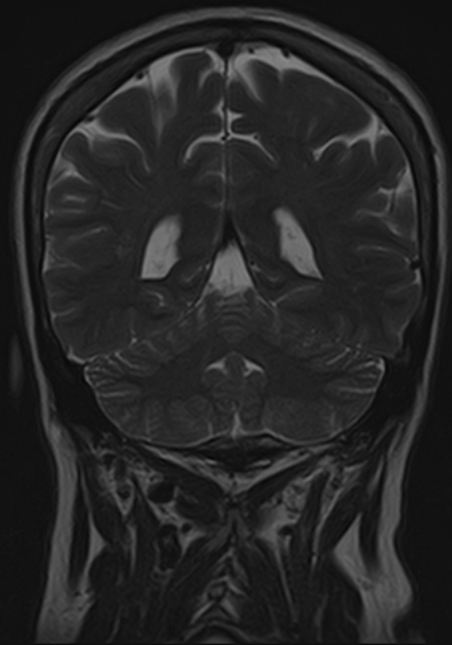

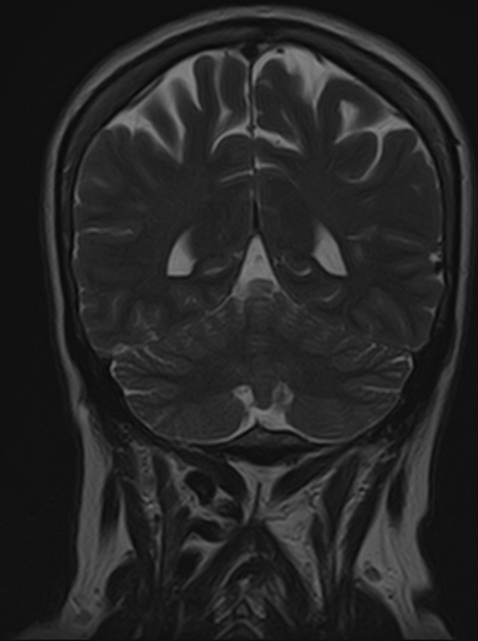

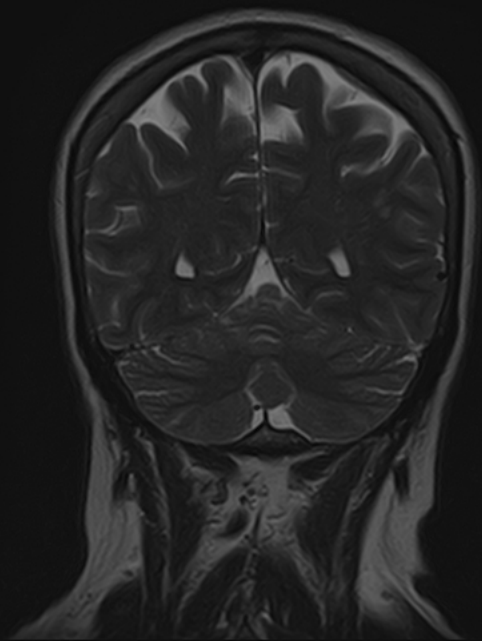

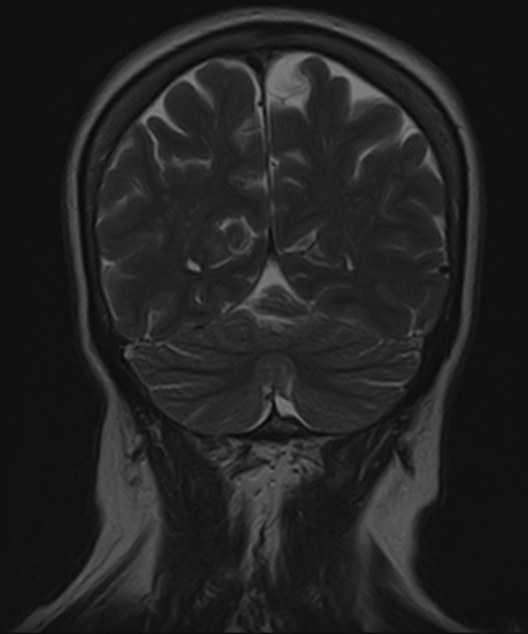

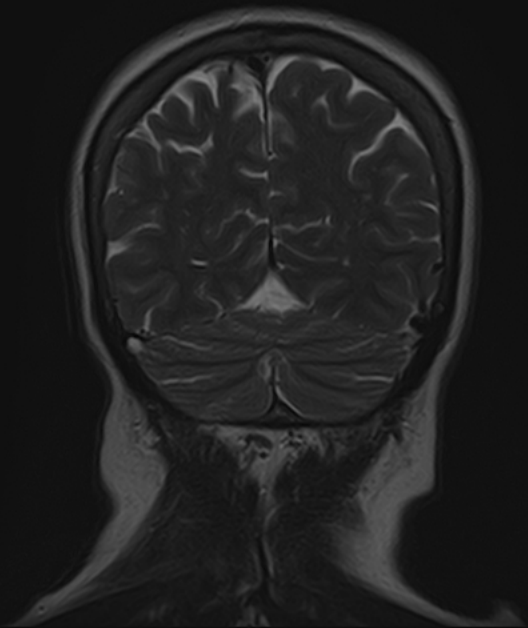

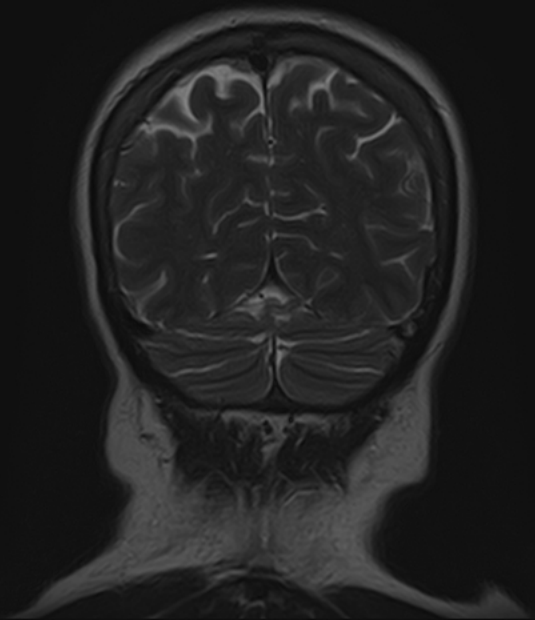

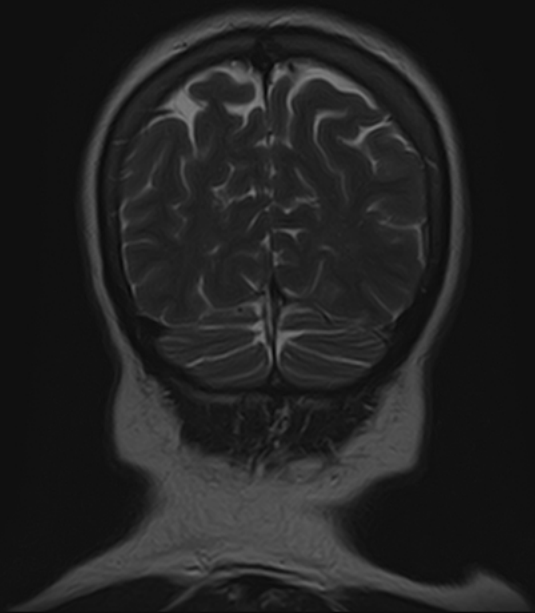

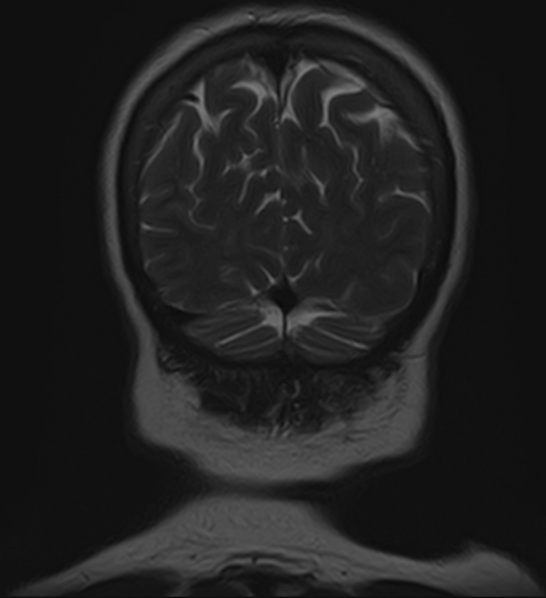

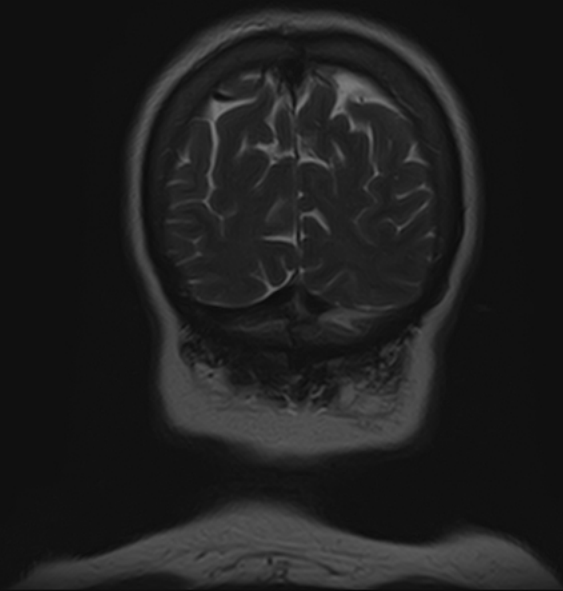

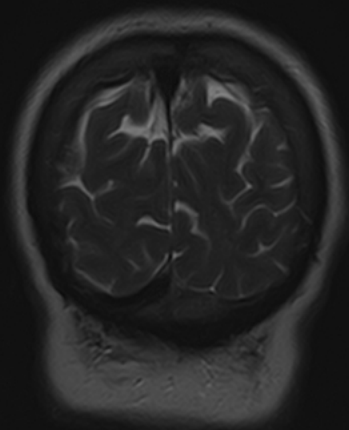

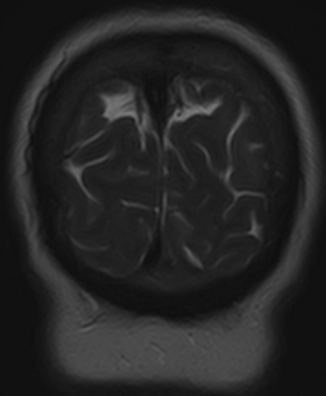

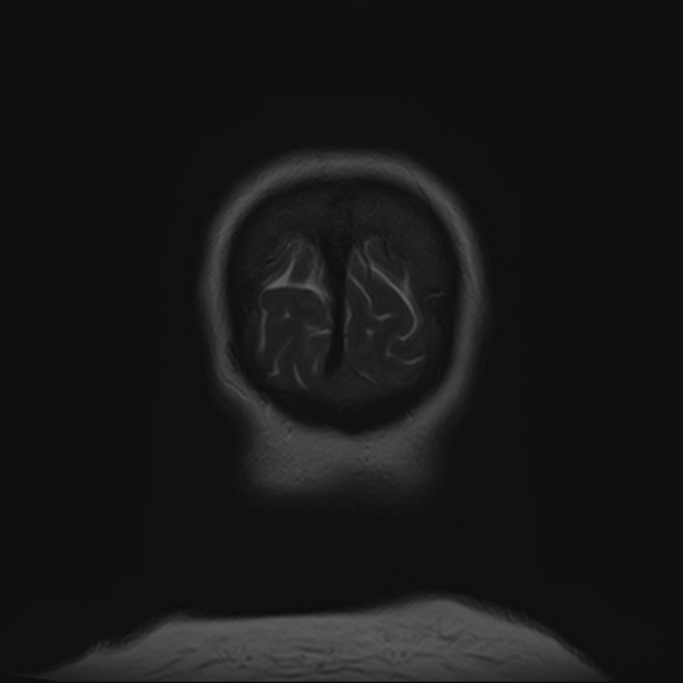

Supplement: Supplementary file 1 [file reports-09-00050-s001.zip › Supplementary materials 1.pdf]
